# The miR-199a/Brm/EGR1 axis is a determinant of anchorage-independent growth in epithelial tumor cell lines

**DOI:** 10.1038/srep08428

**Published:** 2015-02-12

**Authors:** Kazuyoshi Kobayashi, Kouhei Sakurai, Hiroaki Hiramatsu, Ken-ichi Inada, Kazuya Shiogama, Shinya Nakamura, Fumiko Suemasa, Kyosuke Kobayashi, Seiya Imoto, Takeshi Haraguchi, Hiroaki Ito, Aya Ishizaka, Yutaka Tsutsumi, Hideo Iba

**Affiliations:** 1 Division of Host-Parasite Interaction, Department of Microbiology and Immunology, University of Tokyo, Tokyo, Japan; 2 Laboratory of DNA Information Analysis, Human Genome Center, Institute of Medical Science, University of Tokyo, Tokyo, Japan; 3 First Department of Pathology, Faculty of Medicine, Fujita Health University, Aichi, Japan

## Abstract

In epithelial cells, miRNA-199a-5p/-3p and Brm, a catalytic subunit of the SWI/SNF complex were previously shown to form a double-negative feedback loop through EGR1, by which human cancer cell lines tend to fall into either of the steady states, types 1 [miR-199a(−)/Brm(+)/EGR1(−)] and 2 [miR-199a(+)/Brm (−)/EGR1(+)]. We show here, that type 2 cells, unlike type 1, failed to form colonies in soft agar, and that CD44, MET, CAV1 and CAV2 (miR-199a targets), all of which function as plasma membrane sensors and can co-localize in caveolae, are expressed specifically in type 1 cells. Single knockdown of any of them suppressed anchorage-independent growth of type 1 cells, indicating that the miR-199a/Brm/EGR1 axis is a determinant of anchorage-independent growth. Importantly, two coherent feedforward loops are integrated into this axis, supporting the robustness of type 1-specific gene expression and exemplifying how the miRNA-target gene relationship can be stably sustained in a variety of epithelial tumors.

Chromatin remodeling factors play vital roles in epigenetical regulation via genome-wide gene transcription^1^. On the other hand, microRNAs (miRNAs) are post-transcriptional regulatory molecules that are involved in diverse biological processes, including development, differentiation, and homeostasis^2^. Growing evidence indicates that the robustness of gene expression is often supported by coordinated transcriptional and miRNA-mediated regulatory networks^3^,^4^. In addition, improper use of these networks may lead to human diseases such as cancer. However, the interplay between chromatin remodeling factors and miRNA, as well as its biological outcome, is not fully understood in the context of gene regulatory networks common to a wide variety of cell lines.

The human SWI/SNF-A complex (also known as the BAF complex), a member of a family chromatin remodeling factors^5^ composed of about 10 proteins, regulates gene transcription, either positively or negatively. The SWI/SNF complex contains a single molecule of either Brm or BRG1 as ATP-dependent catalytic subunits. Brm and BRG1 regulate target promoters that do not fully overlap and show clear differences in their biological activities^6^,^7^,^8^,^9^. This SWI/SNF complex interacts with various proteins, including transcriptional regulators, through many specific and varied associations with its several subunits. For example, the d4-family proteins DPF2 (REQ) and DPF3a/3b function as efficient adaptor proteins for RELB/p52^10^ and RELA/p50^11^ dimers to induce SWI/SNF-dependent NFκB target genes.

In terms of human cancers, we and other groups have reported that Brm is frequently undetectable in various cancer cell lines^12^, and in primary tumors of the lung^13^, stomach^14^, and prostate^15^. We found in nuclear run-on transcription assays that a functional *Brm* gene was present and actively transcribed in all of the Brm-deficient cancer cell lines tested^12^,^16^, indicating that Brm expression is largely suppressed by post-transcriptional gene silencing. Brm was later shown to be efficiently targeted by both miR-199a-5p and miR-199a-3p^17^. In addition, Brm acts as a potent negative regulator of endogenous *EGR1* gene expression. EGR1 activates the *miR-199a (2)* gene locus, which is mainly responsible for the biogenesis of mature miR-199a-5p and -3p in these cancer cell lines. Overall, these findings suggest that, in the cell lines examined, Brm and miR-199a form a robust double-negative feedback loop that includes EGR1^17^. By examining a panel of human cell lines that were derived from a wide variety of cancer tissues, we found that they tend to fall into either of the steady states, miR-199(−)/Brm(+)/EGR1(−) cells and miR-199a(+)/Brm(−)/EGR1(+) cells^17^, denoted hereafter as type 1 and type 2, respectively. These regulatory networks may explain why variable (either higher or lower) expression of miR-199a-5p/-3p^18^ or EGR1^19^ has been reported among many carcinomas when compared with the normal epithelial tissues from which they originated.

In the early stage of our current study, we noticed clear differences in the biological properties between type 1 and type 2 cells: all of the type 1 cell lines tested (8 lines), but no type 2 cell lines (4 lines), could grow in soft agar, providing us with an unprecedented opportunity to unravel the robust regulatory networks involved in anchorage-independent growth common to these cancer cell lines. Of course, the gene expression patterns of each cancer cell line would be expected to be largely cell line-specific and dependent on a wide variety of factors, including the originating tissue type, mutated genes, and pathological properties, such as the tumor stage. However, in our current study, we speculated that epithelial tumors would share regulatory networks that control their basic biological activities. In addition, we hypothesized that several genes would be specifically expressed in type 1 cancer cells, but not in type 2, and, further, that some of them would be crucial for their anchorage independency. Here, we have identified several genes specifically expressed in type 1 cells and show that single knockdown of some of these genes is sufficient to suppress the colony-forming activity of type 1 cells in soft agar. We further examined the underlying molecular mechanisms of the all-or-none regulation of these type 1-specific genes in the two cell types, leading to the identification of two coherent feedforward loops associated with the miR199a/Brm/EGR1 axis. We finally present evidence that these type-specific gene expression patterns can be recapitulated in tumor some lesions of non-small-cell lung carcinomas (NSCLCs).

## Results

### Type 1, but not type 2, cells grow efficiently in soft agar

**Figure 1:**
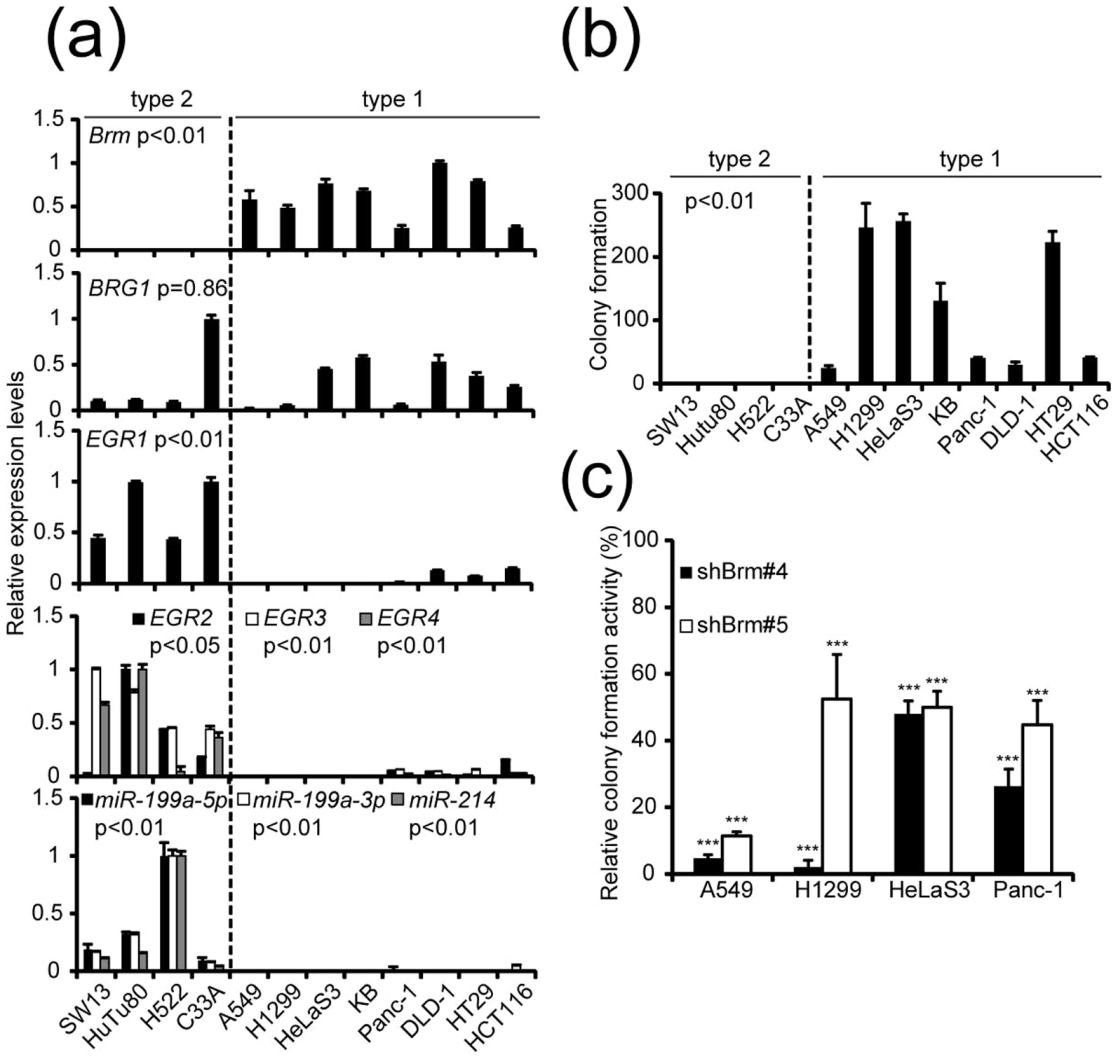
Basic properties of 12 human cell lines originating from various epithelial tumors. (a) Relative expression levels of *Brm*, *BRG1*, *EGR1, EGR2, EGR3*, and *EGR4* mRNA and mature miR-199a-5p, -3p, and miR-214 were determined by quantitative RT-PCR. The data represent the means ± S.D. (n = 3). (b) Numbers of colonies formed in soft agar by each cell line. Numbers of colonies (more than 150 μm in diameter) in soft agar were counted 21–28 days after 1,000 cells were seeded in 60-mm plates. The data represent the means ± S.D. (*n* = 3) In a–b, P values were determined using Mann-Whitney test. (c) Four type 1 cell lines transduced with retroviral vectors expressing shBrm (#4 or #5) or shCre#4 (negative control) were seeded as in b. Colony numbers of shBrm-expressing cells were compared with those of shCre#4-expressing cells and the ratio was shown as a percentage. The data represent the means ± S.D. (*n* = 4). Asterisks indicate P value, compared with those transduced with shCre#4. *P<0.05, **P<0.01, ***P<0.001.

For type 1 and type 2 cells, we chose 12 cell lines (8 for type 1 and 4 for type 2) originating from various human epithelial tumors (Fig. 1a). To ensure that we only examined cell lines originating from epithelial tumors, PA-1 (originating from tridermic teratocarcinoma), MDA-MB435 (recently discovered to originate from melanoma), and HEK-293FT (originating from human embryonic kidney) were removed from the panel of cancer cell lines previously used for categorization^17^. In addition, cell lines from one pancreatic (Panc-1) and three colon cancers (DLD-1, HT29, and HCT116) were added to the current analysis.

The results of a series of quantitative reverse transcription-polymerase chain reaction (RT-PCR) experiments (Fig. 1a) confirmed our previous observations that epithelial tumor cell lines can be classified into two types according to the expression levels of Brm, EGR1, and miR-199a; type 1 cells specifically express *Brm* mRNA, whereas expressions of *EGR1* mRNA and miR-199a-5p and -3p, as well as miR-214, which is also generated from the *miR-199a (2)* gene locus, are restricted to type 2 cells. Notably, like *EGR1*, *EGR2*, *EGR3*, and *EGR4*, which are the other members of EGR family gene and which recognize the same DNA sequence^20^, were shown to be type 2-specific (Fig. 1a) by the parallel analysis. This might indicate EGR2, EGR3, and EGR4 are involved in the miR-199a/Brm axis in a similar manner to EGR1.

After several preliminary comparative analyses between type 1 and type 2 cells, we noticed clear differences in terms of anchorage-independent growth. type 1 cells formed 25–300 colonies (more than 150 μm in diameter) in soft agar when 1,000 cells were seeded per 60-mm plate and kept for 21–28 days (Fig. 1b). None of the four type 2 cells formed clear colonies in the same conditions. Notably, all of the type 2 cancer cell lines tested—SW13^21^, HuTu80^22^, H522^23^ and C33A^24^—have shown clear tumor-forming activity in mouse xenograft models.

To test whether the anchorage-independent growth of type 1 cells requires Brm, we performed Brm knockdown experiments in several type 1 cells—A549, H1229, HeLaS3, and Panc-1—using a set of retroviral vectors containing shBrm (#4 and #5). We confirmed that all shBrm vectors significantly suppressed the levels of *Brm* mRNA and its product (see below) and that cells transduced with these vectors reduced colony-forming activity in soft agar when compared with that of negative control cells transduced with the shCre#4 vector (Fig. 1c). These results reveal the pivotal role of Brm in anchorage-independent growth in type 1 cells. In several cases, strong suppression in colony formation in soft agar was observed by Brm knockdown, whereas the same culture grow normally when kept in monolayer culture (A549 cells expressing shBrm#5 and HeLaS3 and Panc-1 cells expressing either shBrm#4 or #5, Supplementary Fig. 1). In the case of H1299, however, we cannot exclude the possibility that reduction in the growth rate of monolayer culture partly contributed reduction in anchorage-independent growth. These findings provided us with an excellent opportunity to uncover the critical genes required for anchorage-independent growth of type 1 cells and suggested that these candidate genes would be expressed in a type 1-specific manner.

### Several genes are preferentially expressed in type 1 cells

**Figure 2:**
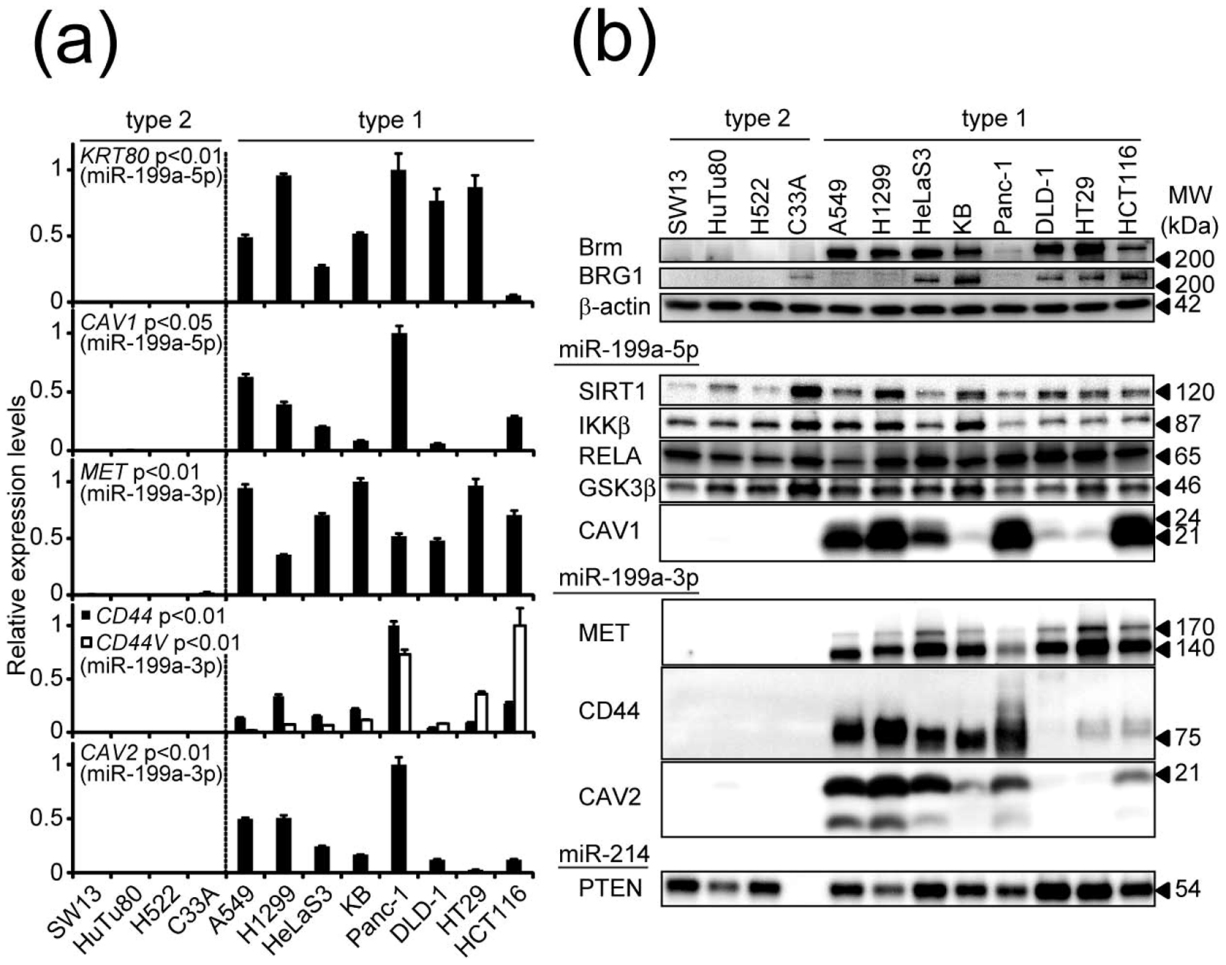
Detection of five type 1-specific genes. (a) Relative expression levels of five type 1-specific genes—*CAV1*, *KRT80*, *MET*, *CD44* (all transcripts and variant types v8–v10), and *CAV2—*in 12 cancer cell lines were determined by quantitative RT-PCR. The data were normalized by taking the highest levels as 1.0. The data represent the means ± S.D. (*n* = 3). P values were determined using Mann-Whitney test. (b) Protein expression levels of Brm, BRG1, and four type 1-specific genes products as well as five type-independent gene products were analyzed by western blotting using 10 gels. The full-length blots were presented in the supplementary Figure 6. β-actin was used as the loading control for each gel. Analysis of KRT80 protein was not possible because of the lack of a specific antibody. Relative expression levels of each protein were quantified in Supplementary Figure 4.

Whereas we know that suppression of the expression of a target protein by a certain miRNA is usually moderate and is not unconditionally retained in steady states, we selected the candidates of type 1-specific genes from various targets of miR-199a-5p (10 genes tested), miR-199a-3p (11 genes tested), and miR-214 (6 genes tested). These target genes were identified in previous reports or were predicted by target prediction algorithms as well as by our own analysis (Supplementary Table 1). Of 32 candidate genes tested by quantitative RT-PCR (Fig. 2a and Supplementary Figs. 2 and 3), *CAV1*^25^ and *KRT80* (both miR-199a-5p target genes) and *CD44*^26^, *MET*^27^, and *CAV2*^28^ (all miR-199a-3p targets) were expressed in most of the type 1 cells but in none of the type 2 cells (Fig. 2a). We also performed FACS analysis for CD44 and MET using H1299 cells (type 1), and found that the entire population showed high levels of both CD44 and MET (data not shorn). We thus designated them as type 1-specific genes. Interestingly, two other epithelial-type keratin genes^29^,^30^, *KRT7*^31^ (an miR-199a-3p target) and *KRT19* (an miR-199a-5p target), were not expressed in any of the type 2 cells but were expressed in some of the type 1 cells (Supplementary Fig. 2 and 3).

The protein expression profiles of most of these type 1-specific genes, as well as four genes whose mRNA was expressed in both types, were examined by western blotting (Fig. 2b) and relative amounts of each protein were quantified (Supplementary Fig. 4). The protein expression profiles were generally very similar to those of the corresponding mRNA (Fig. 2a). Mann-Whitney test further confirmed that these miR-199a target gene products (CD44, MET, CAV1 and CAV2) are specifically expressed in type 1 cells (Supplementary Fig. 4). KRT80 protein was not analyzed because of the lack of a specific antibody. PTEN protein, a well-known miR-214 target, was clearly expressed in all of the cell lines except C33A cells, because C33A have homozygous nonsense mutation of this gene. It is noteworthy that among these type 1-specific proteins, CD44, MET, CAV1 and CAV2 function as plasma membrane sensors and signaling platforms and can be colocalized in caveolae in several physiological conditions^32^.

### A single knockdown of CD44, MET, CAV1, or CAV2 is sufficient to suppress anchorage-independent growth of type 1 cell lines

**Figure 3:**
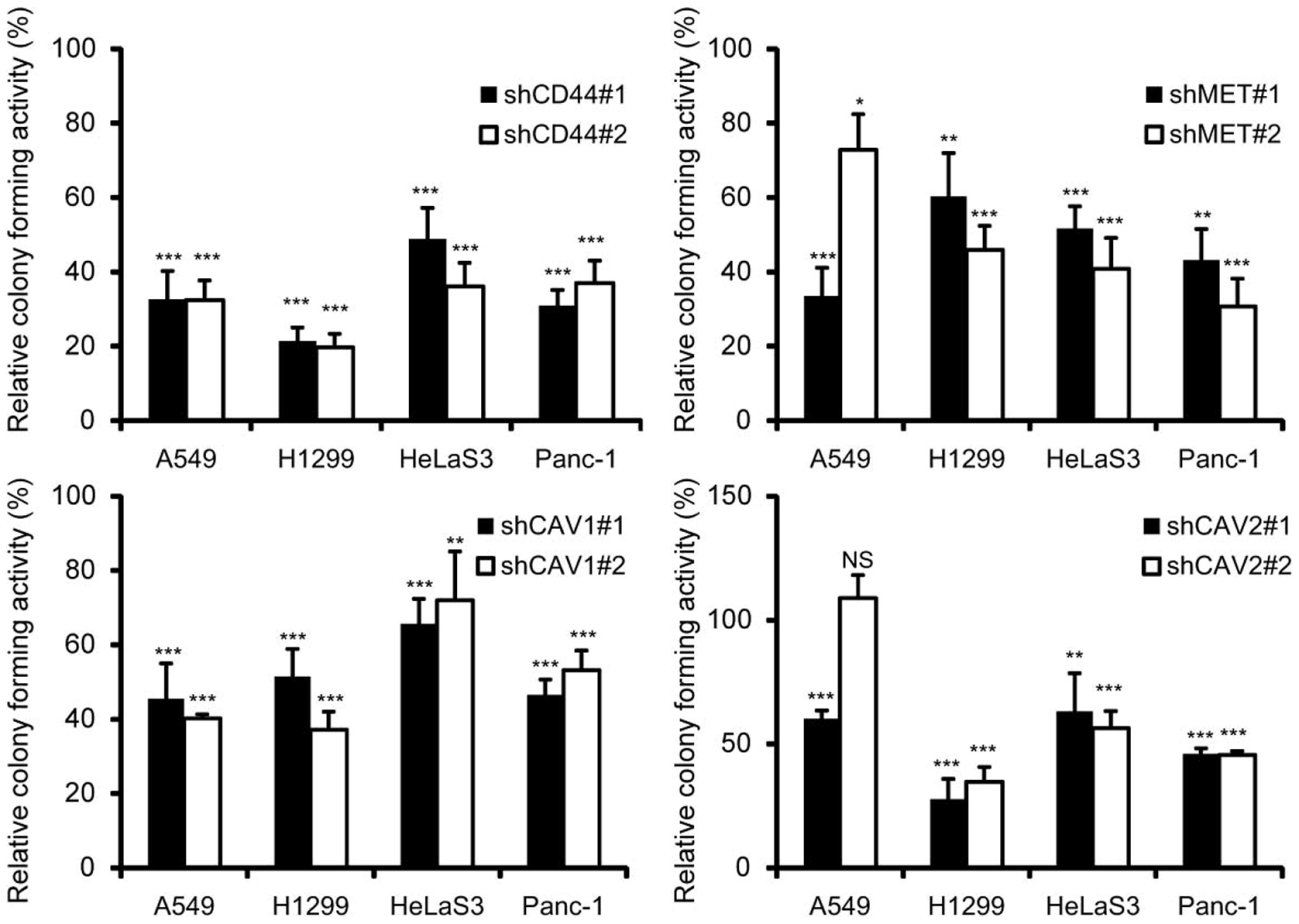
Effect of single knockdown of several type 1-specific genes on anchorage-independent growth of four type1 cell lines. 300-1,000 cells of A549, H1299, HeLaS3 and Panc-1 transduced with retrovirus vectors expressing shCD44 (#1 or #2), shMET (#1 or #2), shCAV1 (#1 or #2), shCAV2 (#1 or #2) or sh Cre#4 (a negative control) were seeded in 60 mm plates. Colony numbers of these shRNA expressing cells were compared to those of shCre#4 expressing cells and the ratio was shown in percentage. The data represent the means ± S.D. (*n* = 4). Asterisks indicate P value, compared with those transduced with shCre#4. NS, not significant. *P<0.05, **P<0.01, ***P<0.001.

To evaluate whether the type 1-specific genes identified above contribute to the anchorage-independent growth of this cell type, we developed pairs of shRNA constructs for *CD44*, *MET*, *CAV1*, and *CAV2* capable of efficiently suppressing their target gene products (Supplementary Fig. 5). Specific knockdown of KRT80 by short hairpin was not possible because there are too many conserved regions among the large *keratin* gene family paralogues. After A549, H1299, HeLaS3, and Panc-1 cells were transduced with the shRNA-expressing retroviral vectors, their colony-forming activity in soft agar was evaluated (Fig. 3). Single knockdown of *CD44*, *MET*, *CAV1*, or *CAV2* efficiently suppressed colony formation compared with negative control cells expressing shRNA for *Cre#4*. As an exception, the colony number of A549 cells expressing shCAV2#2 was slightly increased, probably due to off-target effects. Knockdown of *CD44*, *MET*, *CAV1*, and *CAV2* did not significantly affect cell growth. These results indicate that all of the four type 1-specific genes tested significantly contribute to the anchorage-independent growth of these type 1 cell lines.

### All-or-none expression patterns of some type 1-specific genes in the cell line panel are supported by two coherent feedforward loops that associate with the axis

Given that miRNA usually suppresses its target protein in a modest manner, it might be somewhat unexpected that some miR-199a targets were regulated in an all-or-none manner between type 1 and type 2 cells (Fig. 2a,b). We speculated that this all-or-none phenomenon could be reflecting regulation by the molecular switch through miR-199a/Brm/EGR1 axis, where Brm and miR-199a expressions manifest a mutually exclusive pattern. Therefore, we first tested whether these type-1 specific genes, *CD44*, *MET*, *CAV1*, *CAV2*, and *KRT80* genes are under the positive control of the Brm-type SWI/SNF complex, for which type 1 cells are competent.

**Figure 4:**
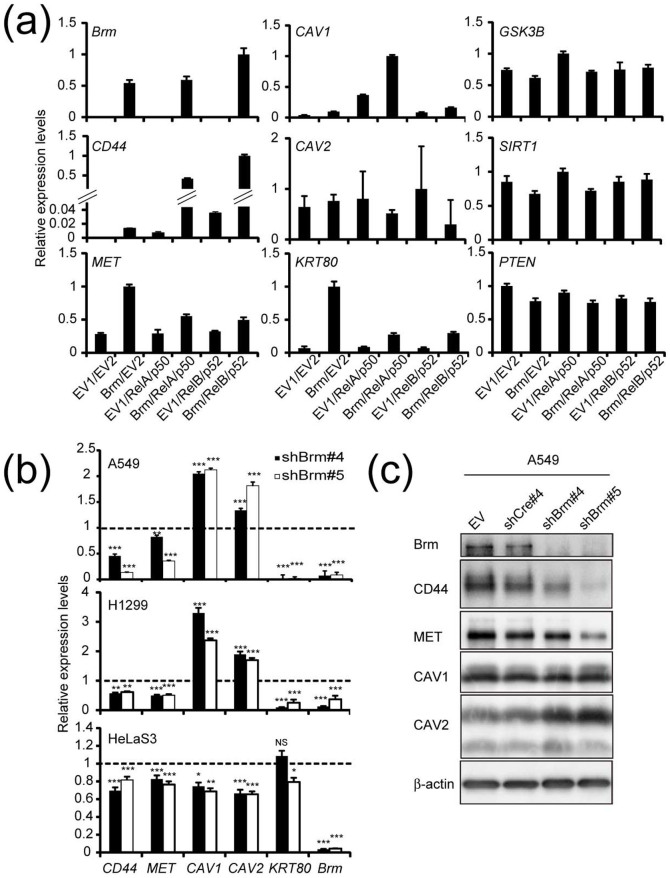
*Brm* is required for the expression of some type 1-specific genes. (a) Expression of *Brm* and type 1-specific (*CD44*, MET, *CAV1*, *CAV2*, and *KRT80*) and non-type-specific (*GSK3β*, *SIRT1* and *PTEN*) genes in SW13 cells transfected with a Brm expression vector or an empty vector (EV1;pCAG-IG) with or without NFκB dimers (RelA/p50, RelB/p52) or another empty vector (EV2;pRK5). The highest expression level was taken as 1.0. The data represent the means ± S.D. (*n* = 3). (b) Relative expression levels of *CD44* (all transcripts), *MET*, *CAV1*, *CAV2*, and *KRT80* as well as *Brm* mRNA in three cell lines of type 1 cells transduced with shBrm-expressing retroviral vector. The expression levels of cells transduced with shCre#4-expressing vector was taken as 1.0. The data represent the means ± S.D. (*n* = 3). Asterisks indicate P value, compared with those transduced with shCre#4. NS, not significant. *P<0.05, **P<0.01, ***P<0.001 (c) Protein analysis of the parallel A549 cultures prepared as shown in b. β-actin was used as the internal control. The full-length blots were presented in the supplementary Figure 7.

To test whether type 2 cells can induce type 1-specific genes when Brm is exogenously introduced, we transfected SW13 cells (type 2) with Brm expression plasmid or empty plasmid (EV1). In these experiments, some parallel cultures were cotransfected with the expression plasmids for representative NFκB dimers—RELA/p50 (canonical pathway) and RELB/p52 (noncanonical pathway)—or empty plasmid (EV2) to determine whether the activation is enhanced by NFκB dimers. As shown in Fig. 4a, *CD44*, *MET*, *CAV1*, and *KRT80* mRNA were induced by Brm, as judged by quantitative RT-PCR, although the Brm induction effect varied among the genes. *CD44* and *CAV1* mRNA levels were increased by cotransfection with NFκB dimers and Brm, whereas expression of *MET* gene was not NFκB dependent at all. *CD44* expression was more strongly dependent upon the noncanonical dimer RelB/p52 than RelA/p50, consistent with a recent report^33^. *CAV1* expression was further increased by cotransfection with RelA/p50 and Brm but was also significantly induced by RelA/p50 alone. Therefore, *CAV1* would only require Brm for its full expression. On the other hands, the expression levels of *GSK3β* and *Sirt1* (miR-199a-5p targets), and *PTEN* (a miR-214 target), which did not show type 1-specific expression patterns, were not affected by high levels of either Brm or NFκB in SW13 cells (Fig. 4a). Next, A549, H1299, and HeLaS3 cells (type 1) were transduced with retroviral vector encoding Brm shRNA, and the steady-state expression of *CD44*, *MET*, *CAV1*, *CAV2*, and *KRT80* was evaluated by quantitative RT-PCR (Fig. 4b). The levels of *CD44*, *MET*, and *KRT80* mRNA were suppressed to various extents by Brm knockdown. Western blot analysis of parallel A549 cultures also indicated that *CD44* and *MET*, but not *CAV1* and *CAV2*, required Brm for expression (Fig. 4c)^7^. This strong Brm-dependency of CD44 expression is consistent with the previous report that assorted tissues from Brm null/BRG1-positive mice lack CD44 expression. Overall, these findings indicated that genes that are suppressed by miR-199a and simultaneously require the Brm-type SWI/SNF complex for efficient expression show distinct expression patterns: expression in type 1 cells but no expression in type 2 cells. But *CAV1* and *CAV2* expression failed to show clear Brm dependency in A549 and H1299 cells.

**Figure 5:**
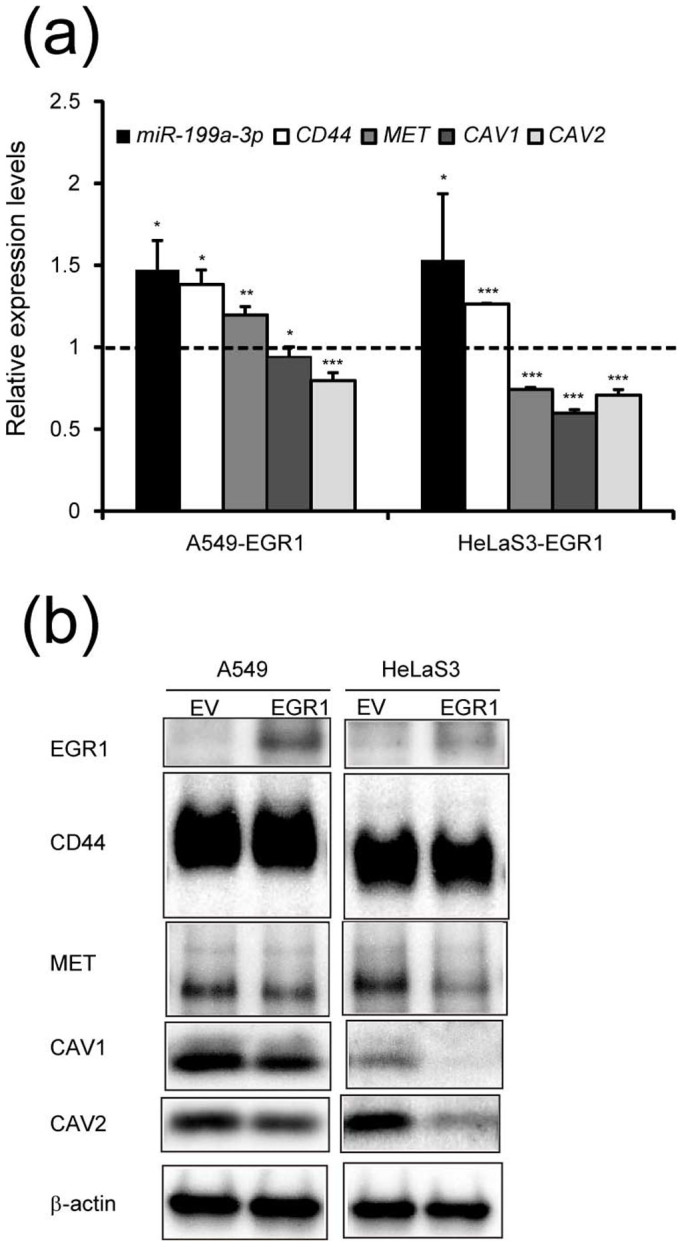
Effects of exogenous EGR1 expression in type 1 cells. (a) Relative expression levels of miR-199a-3p and type 1-specific mRNAs were determined by quantitative RT-PCR in A549 and HeLaS3 cells which were transduced with retroviral vectors expressing EGR1. The expression levels of cells transduced with empty vector was taken as 1.0. The data represent the means ± S.D. (*n* = 3). Asterisks indicate P value, compared with those transduced with empty vector. *P<0.05, **P<0.01, ***P<0.001 (b) Analysis of type 1-specific gene products and EGR1 in the parallel cultures prepared in a by western blotting. β-actin was used as the loading control. The full-length blots were presented in the supplementary Figure 8 and relative expression levels of each protein including two additional sets of blots were quantified in Supplementary Table 2.

We next tested whether type 1-specific genes are under the negative control of EGR1. When HeLaS3 and A549 cells were stably transduced with EGR1-expressing retrovirus, endogenous miR-199a-3p levels were elevated as expected from the axis (Fig. 5a). In HeLaS3, levels of *MET*, *CAV1* and *CAV2* mRNA (Fig. 5a) and their gene products (Fig. 5b and Supplementary Table 2) were reduced by exogenous EGR1 expression. In the case of A549 cells, slight reduction of *CAV1* and *CAV2* mRNA and reduction of MET, CAV1 and CAV2 proteins were observed (Fig. 5ab and Supplementary Table 2). These results are consistent with previous reports indicating that *MET*^34^ and *CAV1*^35^ genes are negatively regulated by EGR1: the *MET* and *CAV1* promoters have one and three EGR/SP-1 binding sites, respectively. We also found EGR1 binding sites on the *CAV1*, *CAV2* and *MET* promoter regions (from -1600 to +500bp of TSS) by using ChIP-seq data obtained by ENCODE. These results suggest that *CAV1/2* and possibly *MET* are specifically expressed in type 1 cells by evading transcriptional suppression by EGR1 proteins and also post-transcriptional suppression by miR-199a-5p/3p.

**Figure 6:**
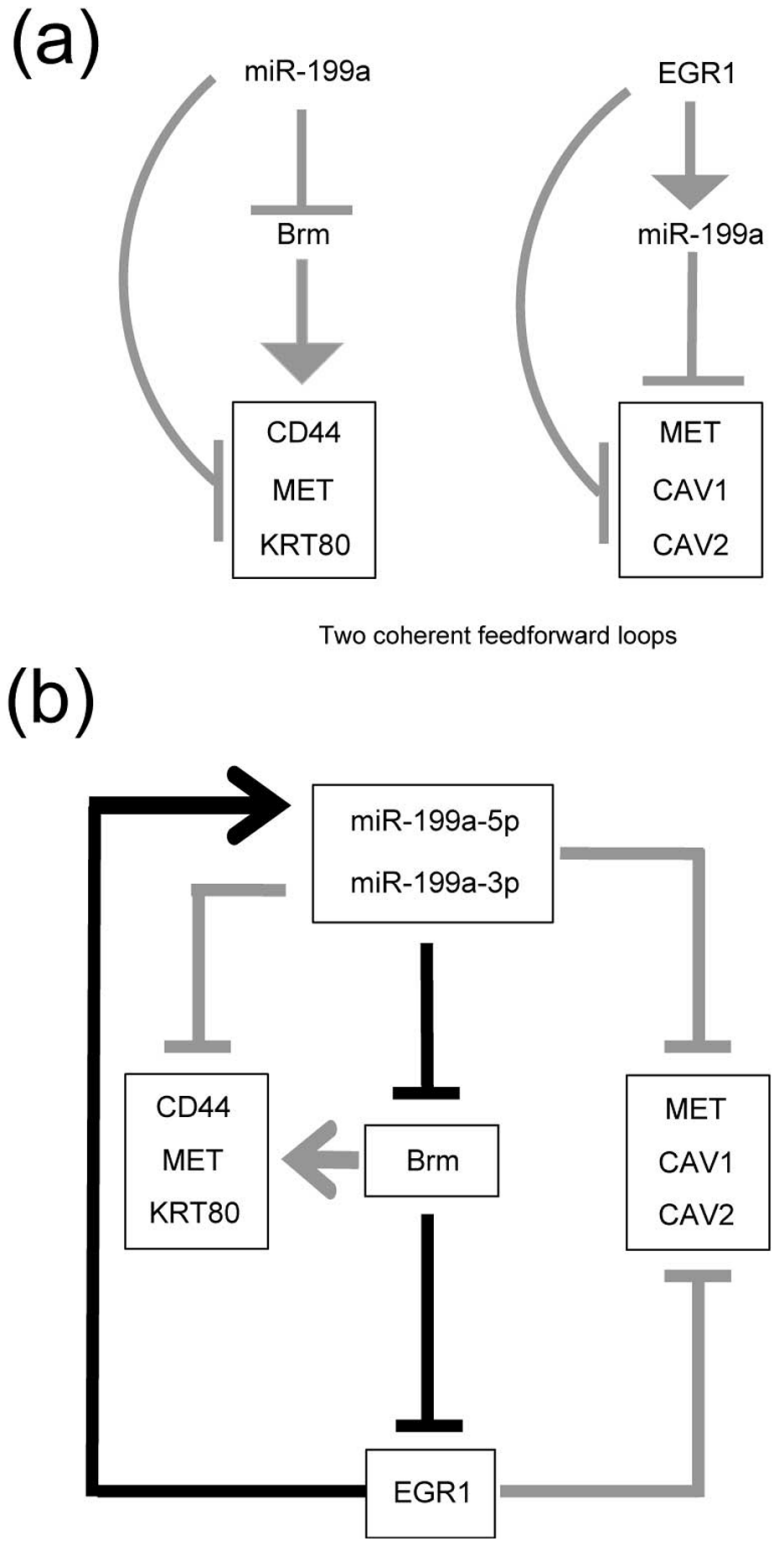
Models of the regulatory networks operating in various epithelial tumor cell lines. (a) Two feedforward loops that function to establish type 1-specific gene expression in an all-or-none manner. (b) A double-negative feedback loop (indicated by black arrows) is integrated by the two feedforward loops (indicated by gray arrows) shown in a.

Overall, these results suggest that there are at least two feedforward loops. One is composed of miR-199a-5p/3p, Brm and CD44, MET and KRT80 (Fig. 6a left) and another is composed of EGR1, miR-199a-5p/-3p and CAV1, and CAV2 (and possibly MET) (Fig. 6a right). Type 1-specific genes would be regulated in an all-or-none manner by either of these two feedforward loops that associate with the robust miR-199a/Brm/EGR1 axis that dictates cancer cell lines to either of the steady states, [miR-199(−)/Brm(+)/EGR1(−)] and [miR-199a(+)/Brm(−)/EGR(+)] (Fig. 6b).

### The miR-199a/Brm/EGR1 axis persists in an extended panel of cell lines originating from epithelial tumors

**Figure 7:**
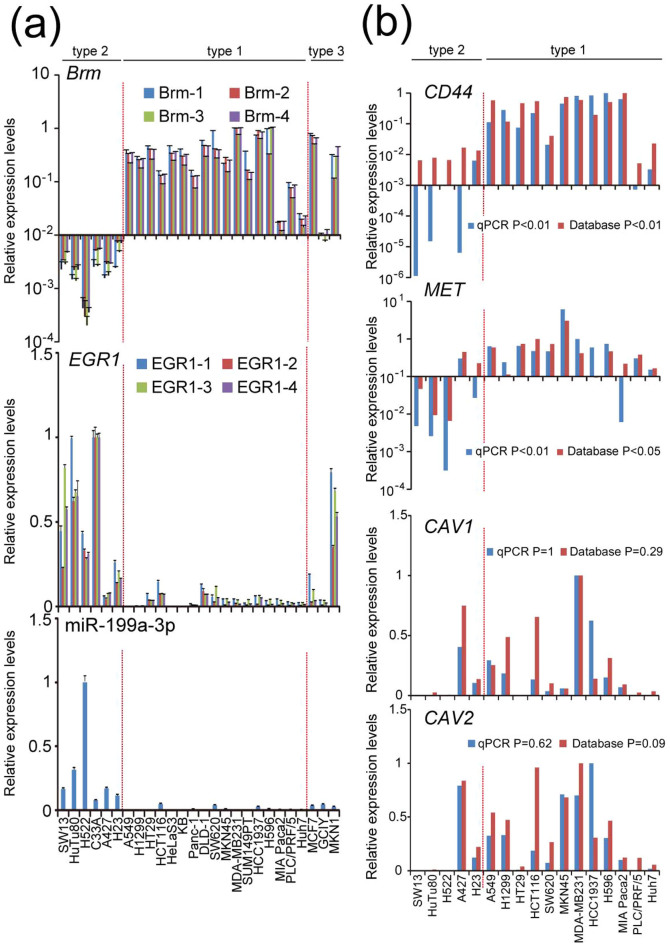
Gene expression analysis on an extended epithelial tumor cell line panel. (a)Expression profiles of *Brm* and *EGR1* mRNA and mature miR-199a-3p RNA of 26 cell lines, as determined by quantitative RT-PCR. Three additional primer pairs were designed and for *Brm* and *EGR1* mRNA quantification other than used in Fig. 1 (Brm-1, EGR1-1). The relative expression levels are shown by taking the highest as 1.0. Two red vertical break lines indicate the boundary among each type cell lines. Detailed criteria for type1-3 cells were indicated in Supplementary Table 3. (b)Expression profiles of *CD44*, *MET, CAV1 and CAV2* mRNA of 17 cell lines, determined by quantitative RT-PCR (blue bars) or obtained from Sanger database (red bars).

Because our panel of cancer cell lines used for the development of the cell line typing was limited to 14 cell lines, we intended to increase the number of cell lines by directly performing quantitative RT-PCR of *Brm* mRNA (using totally 4 PCR primer pairs), *EGR1* mRNA (using totally 4 PCR primer pairs) and miR-199a-3p by adding 12 new cell lines using the same experimental protocol as used for Fig. 1a (Fig. 7). The 4 independent PCR primer pairs for *Brm* or *EGR1* gave essentially the same expression profile, respectively (Fig. 7a). Out of the total 26 cell lines, 23 could be categorized as either type 1 (17 lines) or type 2 (6 lines), according to the criteria shown in Supplementary Table 3. The remaining 3 lines, which were originated from gastric carcinomas and mammary tumors, cannot be categorized into either type 1 or type 2 (designated type 3). These results indicate that the miR-199a/Brm/EGR1 axis is largely retained in variety of epithelial tumor cell lines.

Since microarray data of *Brm* and *EGR1* mRNA for 17 among these cell lines categorized as type 1 and type 2 were available from Sanger database (Genomics of Drug Sensitivity in Cancer http://www.cancerrxgene.org), and their expression profiles obtained from the database was compared with those of the qRT-PCR data shown in Fig. 7a (Supplementary Fig. 9). We found that the expression profiles of *Brm* and *EGR1* are not correlates well between them. Since *Brm* mRNA levels of even Brm-deficient cell lines such as SW13, H522, C33A, A427, and H23—previously reported by our^6^,^12^ and other groups^7^,^36^ by RT-PCR or Northern blotting analysis—were significantly high according to Sanger database, there would be limitations in microarray data to estimate mRNA levels of such transcriptional regulatory genes as *Brm* and *EGR1* accurately.

Since we found expression profiles of *CD44*, *MET*, *CAV1* and *CAV2* mRNA by our qRT-PCR and those obtained from Sanger Database are correlated well, we showed both of them in Fig. 7b. The expression levels of *CD44* and *MET* were high in type 1 cell lines, whereas they were mostly undetectable in the type 2 cell lines even in these extended panels, and specific *CD44* and *MET* expression in type 1 cells were statistically supported in both data of qRT-PCR and the Database. *CAV1* and *CAV2* expression was not detected in most type 2 cells with a clear exception of A427. Because of this, type 1 specific expression of *CAV1* and *CAV2* was not supported statistically. Relatively low *EGR1* expression in A427 among type1 cells (Fig. 7a, Supplementary Table 3) might partly explain this exception.

### Expression patterns observed in type 1 or type 2 cell lines are recapitulated in some cancer lesions of NSCLCs

We finally examined whether the distinct expression patterns observed between the two cell types are reflected in human primary tumors. Since the cell lines originating from NSCLCs in the cell line panel used here can be categorized as both type 1 and type 2 (Supplementary Table 3), we pathologically analyzed surgically resected, formalin-fixed, paraffin-embedded tissues from human cancer lesions of NSCLCs. Among NSCLCs, we especially focused upon squamous cell carcinoma (SCC), because in this type of cancer, we can easily understand activity of proliferation or the status/direction of differentiation two-dimensionally in the histological section. After preparing sequential thin sections of total 21 SCC cases, they were immunohistochemically stained with antibodies against Brm, CD44, MET, and CAV1 and also probed for miR-199a-5p by *in situ* hybridization and interrelationships among their expression patterns in the coincident area of the each section were analyzed by comparing lower and higher differentiation status.

**Figure 8:**
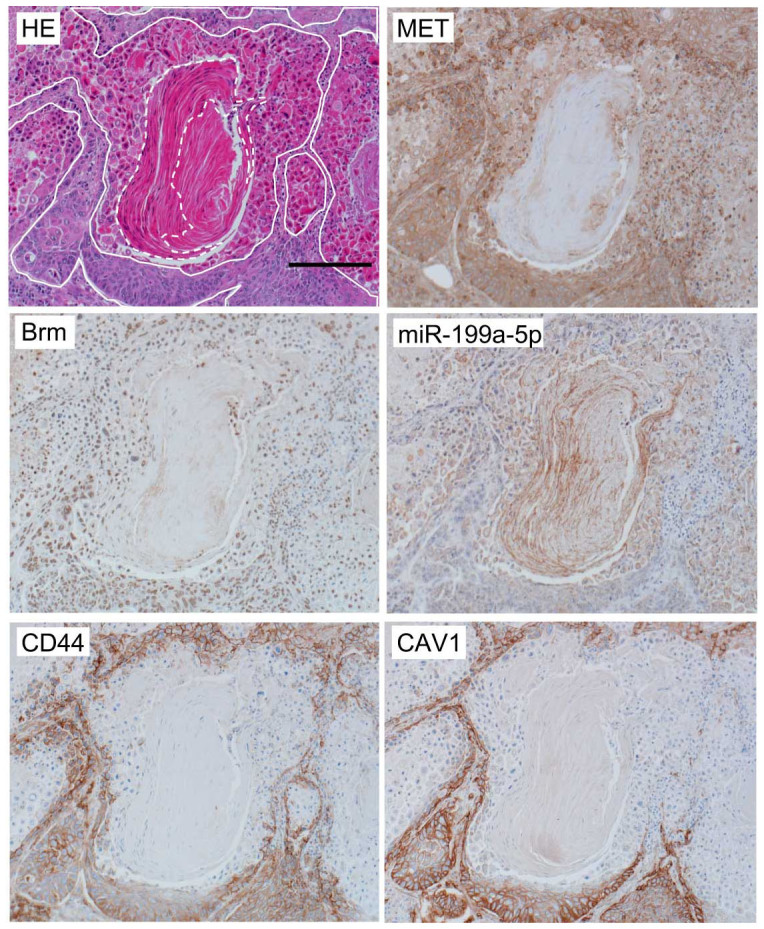
SCC categorized as NSCLC analyzed by *in situ* hybridization for miR-199a-5p or immunohistochemistry for Brm, CD44, MET, CAV1. The bar indicates 200 μm. In the HE staining slide, less differentiated cells are surrounded by white solid lines and highly differentiated cells which still retain cellular nuclei at the periphery of a cancer pearl are shown by a white broken line, respectively.

In the area of lower differentiation status where cancer cells are crowded by the active proliferation and have increased nuclear/cytoplasmic ratio without keratinization, we clearly observed a Brm^+^, CD44^+^, MET^+^, and CAV1^+^ phenotype in almost all cases. In some of these areas, expression of miR-199a was undetectable as shown in Fig. 8 (surrounded by solid line), which recapitulates the expression patterns of type 1 cells. However, in the other areas expressing these 4 proteins, we detected also miR-199a expression, indicating expression heterogeneity in cancer legions.

As for the areas of highly differentiation status, we observed them in many so-called cancer pearls in 4 cases of SCC, where cancer cells are sparse with large cytoplasm. Even in cancer pearls, significant population at the periphery retains clearly recognized nuclei indicating that cells are still alive, but in the central regions, cells are gradually losing their nuclei on their process of keratinization. We detected a Brm^−^, CD44^−^, MET^−^, and CAV1^−^, and miR-199a^+^ phenotype in all of the cancer pearls where the cell retained nuclei, which recapitulated that of type 2 cells (Fig. 8 within the broken line). In the area between solid line and the cancer pearl in Fig. 8, where tumor cells assumed intermediate differentiation status, these 4 proteins and miR-199a were weakly expressed with various extents. At least in these regions, tumor cells might be undergoing changes from the type 1 cells into the type 2 cells through the process of cellular differentiation.

## Discussion

Using 12 cell lines that were strictly derived from human epithelial tumors, we can confirm the findings of our previous report that these cells can be classified into type 1 [mir-199a(−)/Brm(+)/EGR1(−)] (8 lines) or type 2 [miR-199a(+)/Brm(−)/EGR1(+)] (4 lines) cells. In our current study, we were able to efficiently identify the type 1-specific genes by setting the reported miR-199a and miR-214 target genes as the candidates. Some of the identified type 1-specific genes (*CD44*, *MET*, and *KRT80*) required *Brm*, whereas others (*CAV1, CAV2* and probably *MET*) required the absence of EGR1 for their efficient expression, indicating that two coherent feedforward loops are formed (Fig. 6a). These two feedforward loops are integrated into the robust double-negative feedback loop forming a regulatory network that functions as an efficient switch that determines the expression levels of these type 1-specific genes in an all-or-none manner (Fig. 6b). Thus, the current situation would be a good example of a network formed by multiple miRNA-mediated feedback and feedforward loops^37^,^38^, which are commonly present in a wide variety of cell lines. Importantly, we observed regions whose expression patterns recapitulated those of type 1 or type 2 cells by pathological analysis of SCC legions of NSCLC tissues. It should be pointed out, however, that there are several lesions whose expression patterns do not belong to either of them. We speculate that in the process of cell line establishment from primary tumors, they would tend to fall into either of steady states, type 1 or type 2 cells.

Among the type 1-specific genes shown here, *CD44*, *MET*, *CAV1*, and *CAV2* alone significantly contributed to anchorage-independent growth of type 1 cells when tested in knockdown experiments using four type 1 cell lines (Fig. 3). Several previous reports indicated that CD44^39^, MET^40^, and CAV1^41^,^42^ are potentially important for colony formation in some epithelial tumor cell lines. We observed that these four genes are all simultaneously suppressed to a marginal level in type 2 cells by the regulatory network shown here, ensuring the anchorage dependency of type 2 cells. Whereas CD44^43^,^44^, MET^45^, CAV1^46^, and CAV2^47^ have their own multiple downstream signaling pathways, their interplay would also contribute to anchorage-independent growth or metastasis^48^,^49^. Importantly, CD44 and MET were reported to co-localize with CAV1 in caveolae^50^, and CAV2 is also localized in caveolae when it forms hetero-oligomers with CAV1^51^. In normal epithelial cells, caveolae function as plasma membrane sensors, responding to changes in extracellular matrix via integrin signaling and also as interacting domains with cytoskeltones^32^. By unregulated expression of these four proteins in type 1 cells, their intimate and coordinated interactions would generate strong downstream signaling preferable to grow in soft agar. Therefore, in normal epithelial cells, we expect miR-199a-5p and -3p would fine-tune caveolin function such as homeostasis for plasma membrane integrity, signaling platforms, cytoskeleton remodeling and cell migration. In addition to overlapping subcellular localizations of these gene products, it should also be pointed out that the genetic loci of *MET*, *CAV1*, and *CAV2* all reside in the fragile chromosomal region, FRA7G at 7q31.2^52^. These regions seems to be neither amplified nor deleted based on the copy number data compiled in CCLE (https://ccle.ucla.edu/); copy number data of eight cell lines (data of SW13, C33A, HeLaS3 and KB are not available) and the copy numbers of this region are distributed from 1.72 to 2.61.

Currently, we cannot fully explain why type 2 cells can form tumors when tested in xenograft models using immunodeficient mice but fail to form colonies in soft agar *in vitro*. Our preliminary analyses indicated that type 2 cells express *TNF-α*, *IL-1α*, or *CCL5*, whereas they do not express Brm-dependent genes, *IL-6* and *IL-8*, whose products support cell-autonomous growth in soft agar^53^. When type 2 cells are introduced into mice, their production of TNF-α, IL-1α, or CCL5 is expected to induce the production of cytokines such as IL-6 and IL-8 in the associated fibroblast-like cells, which might in turn function as paracrine factors in tumor formation.

Stable *Brm* knockdown in type 1 cell lines by short hairpin retroviral vectors reduced the expression levels of several type 1-specific genes when examined within 2 weeks after the transduction, but cloning of these shBrm-expressing cells in culture is usually difficult^54^. These genes are induced by the transient expression of Brm in type 2 cells (Fig. 4a), but type 2 cells stably expressing exogenous Brm are also difficult to establish, as observed by our group^6^ and others^55^. These observations indicate that transition from either type 2 to type 1 or from type 1 to type 2 is inevitably partial and transient, suggesting that both type 1 and type 2 cells are strongly tied to their own state after the loops are established and therefore stable switching to the opposite type would be difficult. This robustness of each type may reflect some aspects of “oncogene addiction”^56^ or “oncomiR addiction”^57^.

The results in our present study reveal that in normal cells, the interplay between chromatin remodeling factors and miRNAs would fine-tune plasma membrane sensors by several motifs including the miR-199a/Brm/EGR1 axis and two feedforward motifs detected here. These motifs, once misapplied during the process of carcinogenesis, would finally fix the cancer cells to extreme steady states, which cannot be easily reversed. We believe our current findings will give us clues to elucidate how the homeostatic balance is abrogated at cancer initiation to establish type 1 or type 2 tumors and how to guide the development of distinct therapeutic strategies in each case.

## Methods

### Cell culture

The following human cell lines were used in this study: SW13 (adrenocortical carcinoma) [SW13(vim-) was used as a subtype of SW13 that is deficient in Brm and BRG1]^16^; HuTu80 (duodenum carcinoma; the previous nomenclature, AZ521, was corrected according to the instructions of the American Type Culture Collection); NCI-H522, A549, and NCI-H1299 (non-small-cell lung carcinoma); C33A and HeLaS3 (cervical carcinoma); KB (recently shown to be a derivative of HeLaS3); Panc-1 (pancreatic carcinoma); and DLD-1, HT29, and HCT116 (colon carcinoma). All cultures were maintained in Dulbecco's modified Eagle's medium containing 10% fetal calf serum. A549, NCI-H522, NCI-H1299, C33A, A549, KB, Panc-1, DLD-1, HT29, and HCT116 cell lines were purchased from the American Type Culture Collection. AZ521 (HuTu80) and HeLaS3 cell lines were obtained from the Cell Resource Center for Biomedical Research, Institute of Development, Aging and Cancer, Tohoku University, Japan.

### Expression vectors

Expression vectors for Brm (pCAG-Brm-IG)^58^ and NF-κB-expressing vectors (pRK5-RelA, -RelB, -p50, and p52)^10^ used in this study have been described previously. To generate EGR1 expressing retrovirus vector, a *Eco*RI-*Not*I DNA fragment of pCMV-SPORT6-EGR1^17^ was inserted to the corresponding cloning site of pMXs-IRES-Puro or -Bla.

### Plasmid preparation for retroviral vectors expressing shRNA

Pairs of oligonucleotides encoding gene-specific short hairpin RNA (shRNA) were synthesized (Supplementary Table 4) and inserted between the *Bbs*I/*EcoR*I sites of pmU6. The pmU6 derivatives shCre#4^59^ [used as negative control (NC)] and shBrm#4^17^ were previously described. These pmU6-based plasmids were digested with *Bam*HI and *Eco*RI and inserted between these sites in pSSSP for the retroviral vectors.

### DNA transfection and preparation of retrovirus

For the transfection of plasmid vectors into cell lines, Lipofectamine 2000 (Invitrogen Corp.) was used in accordance with the manufacturer's instructions. The preparation and transduction of vesicular stomatitis virus-G (VSV-G) pseudotyped retroviral vectors were performed as described previously^10^.

### Quantitative RT-PCR

Total RNA was extracted using a mirVana microRNA Isolation Kit (Ambion). To detect coding gene mRNAs, cDNA was synthesized with a PrimeScript™ RT Reagent Kit with gDNA Eraser (Perfect Real Time) (TaKaRa Bio) in accordance with the manufacturer's instructions. Quantitative (real-time) RT-PCR was performed using a SYBR® Select Master Mix (Applied Biosystems). *GAPDH* was used as an internal control. The primer pairs used are listed in Supplementary Table 4. For the detection of miRNA, miRNA-specific looped RT-primers and TaqMan probes were used as described by the manufacturer's protocol (Applied Biosystems). *RNUB6* RNA was used as an internal control. PCRs were performed in triplicate using a 7300 Real-Time PCR system (Applied Biosystems).

### Western blotting

Total protein extracts were prepared by boiling the cells in SDS sample buffer for 5 min at 95°C. The proteins were then separated by 10% SDS-PAGE and transferred onto Immobilon-P PVDF membranes (Millipore). Immunoblotting was performed by incubating the membrane overnight at 4°C with primary antibodies against the following proteins: Brm (ab15597; Abcam), BRG1 (sc-10768; Santa Cruz), EGR1 (#4153; Cell Signaling), CD44 (#3570; Cell Signaling), MET (#8198; Cell Signaling), CAV1(#3267; Cell Signaling), CAV2 (#8522; Cell Signaling), SIRT1 (sc-74465; Santa Cruz), GSK3β (#12456; Cell Signaling), RELA (ab7971; Abcam), PTEN (#9552; Cell Signaling), IKKβ (#8943; Cell Signaling), and β-actin (sc-47778; Santa Cruz). After three washes with Tris-buffered saline (TBS) containing Tween 20, the membranes were incubated with secondary antibodies [donkey anti-rabbit-horseradish peroxidase (AP182P) and donkey anti-mouse-horseradish peroxidase (AP192P)]; Millipore) for 1 h at room temperature. Signals were detected using ECL reagent (Promega) or ImmunoStar LD (Wako). Amounts of charged protein samples were roughly normalized to β-actin. Relative protein amounts were quantified by Ez-Capture MG (ATTO) using Multi Gauge V3.2 (Fuji Film) software after strict normalization by the internal control, β-actin.

### Immunohistochemical staining

Deparaffinization, endogenous peroxidase inactivation, and antigen retrieval of formalin-fixed, paraffin-embedded clinical tissues and immunostaining of Brm were performed as described previously^17^,^60^. For CD44 (#3570; Cell Signaling), MET (ab51067; Abcam), and CAV1 (sc-894; Santa Cruz) immunostaining, the sections were incubated overnight at room temperature with the corresponding antibody used for western blotting and washed in phosphate-buffered saline. N-Histofine® Simple Stain™ MAX PO (MULTI) (414154F; Nichirei Biosciences Inc.) were then applied to the slides for 30 min at room temperature, followed by three washes in PBS. The reaction products were visualized using a 50 mg/dL 3,3′-diaminobenzidine tetrahydrochloride solution containing 0.003% H_2_O_2_. The immunostained sections were evaluated independently by two pathologists in conjunction with hematoxylin and eosin-stained sections from the same lesions.

### *In situ* hybridization

*In situ* hybridization analysis to detect miR-199a-5p in formalin-fixed, paraffin-embedded sections was performed using LNA-modified oligonucleotide probes as described previously^17^,^60^. Use of the clinical tissue sections in this study was approved by the Fujita Health University ethical review board for human investigation.

### Statistical analysis

Results are presented as means ± S.D. Statistical significance for quantitative RT-PCR assays was determined using a two-tailed Student's t-test. Statistical significance for the differences of a parameter between type 1 and type 2 cell lines was determined using Mann-Whitney test. In both cases, P-values<0.05 were considered statistically significant.

## Author Contributions

Kazuyoshi K., K. Sakurai, and H. Iba, designed all the experiments and wrote the manuscript. Kazuyoshi K. performed and analyzed a large part of the experiments. H.H., S.N., F.S. and Kyosuke K. contributed protein analysis and plasmid construction. K.I., K. Shiogama and Y.T. prepared clinical samples and performed pathological analysis. S.I. conducted database analysis. T.H., H. Ito and A.I. gave important advices for vector construction, protein analysis and cellular preparation. All authors reviewed the manuscript.

## Supplementary Material

Supplementary Information Supplementary Information
