# Supplementary material for: The miR-199a/Brm/EGR1 axis is a determinant of anchorage-independent growth in epithelial tumor cell lines
Source: Sci Rep. 2015 Feb 12;5:8428. doi: 10.1038/srep08428 (PMC4325331; doi:10.1038/srep08428)
Supplement: Supplementary Information [file srep08428-s1.pdf]

Supplementary information

Supplementary Figure 1-9
Supplementary Table 1-4

The miR-199a/Brm/EGR1 axis is a determinant of anchorage-independent growth in epithelial tumor cell lines

Kazuyoshi Kobayashi[1], Kouhei Sakurai[1], Hiroaki Hiramatsu[1], Ken-ichi Inada[3], Kazuya Shiogama[3], Shinya Nakamura[1], Fumiko Suemasa[1], Kyosuke Kobayashi[1], Seiya Imoto[2], Takeshi Haraguchi[1], Hiroaki Ito[1], Aya Ishizaka[1], Yutaka Tsutsumi[3] & Hideo Iba[1]

[1]Division of Host-Parasite Interaction, Department of Microbiology and Immunology
[2]Laboratory of DNA Information Analysis, Human Genome Center, Institute of Medical Science, University of Tokyo, Tokyo, Japan.
[3]First Department of Pathology, Faculty of Medicine, Fujita Health University, Aichi, Japan

Corresponding author:
Hideo Iba, Division of Host-Parasite Interaction, Department of Microbiology and Immunology, Institute of Medical Science, University of Tokyo, 4-6-1 Shirokanedai, Minato-ku Tokyo, 108-8639, Japan
Phone: 81-3-5449-5730, Fax: 81-3-5449-5449,
E-mail: iba@ims.u-tokyo.ac.jp

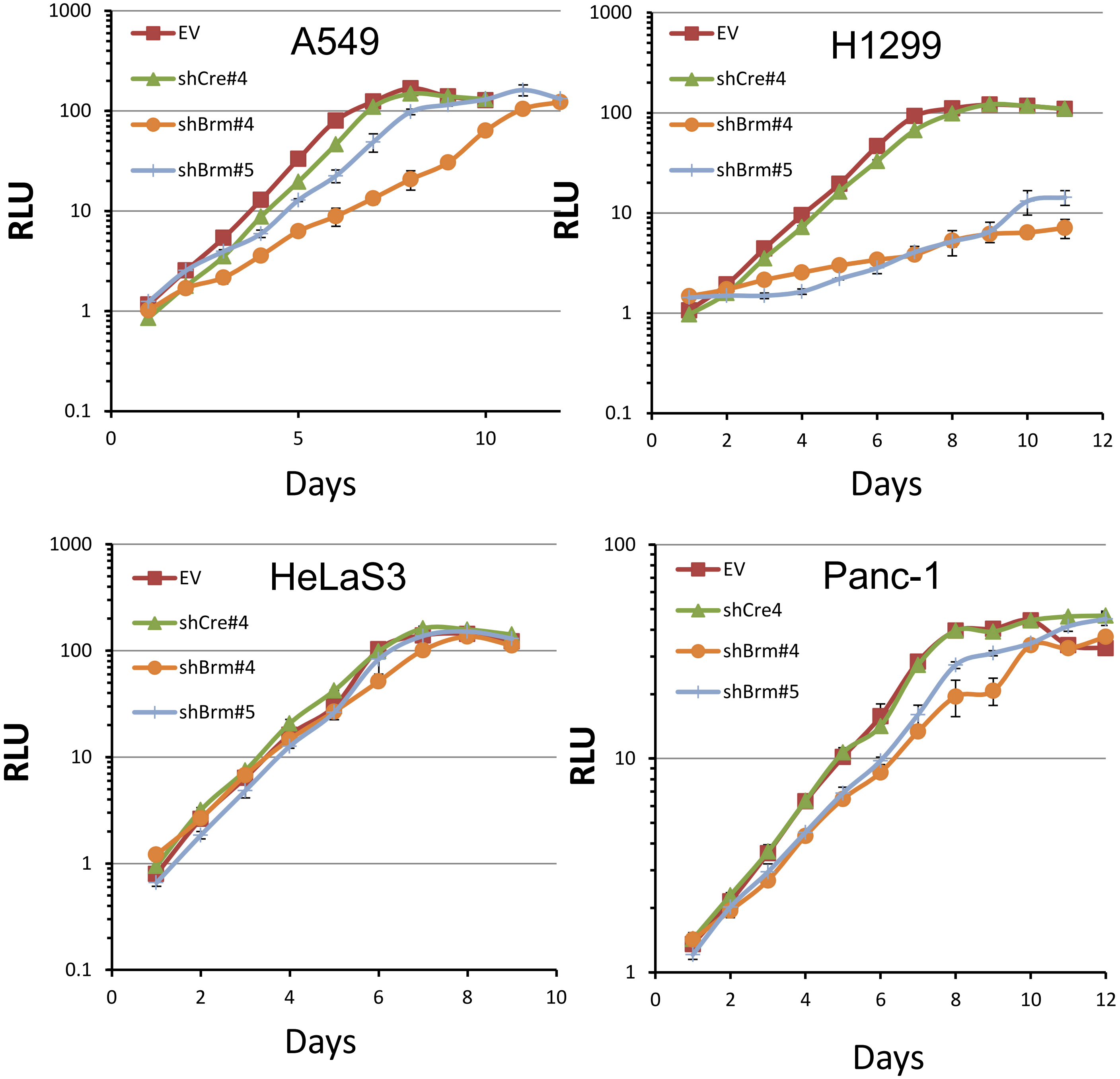

Supplementary Figure 1. Growth curve analysis of four type 1 cell lines that were transduced with retroviral vector expressing shBrm (#4 or #5) or shCre#4 (negative control). EV = transduced with an empty vector (pSSSP).

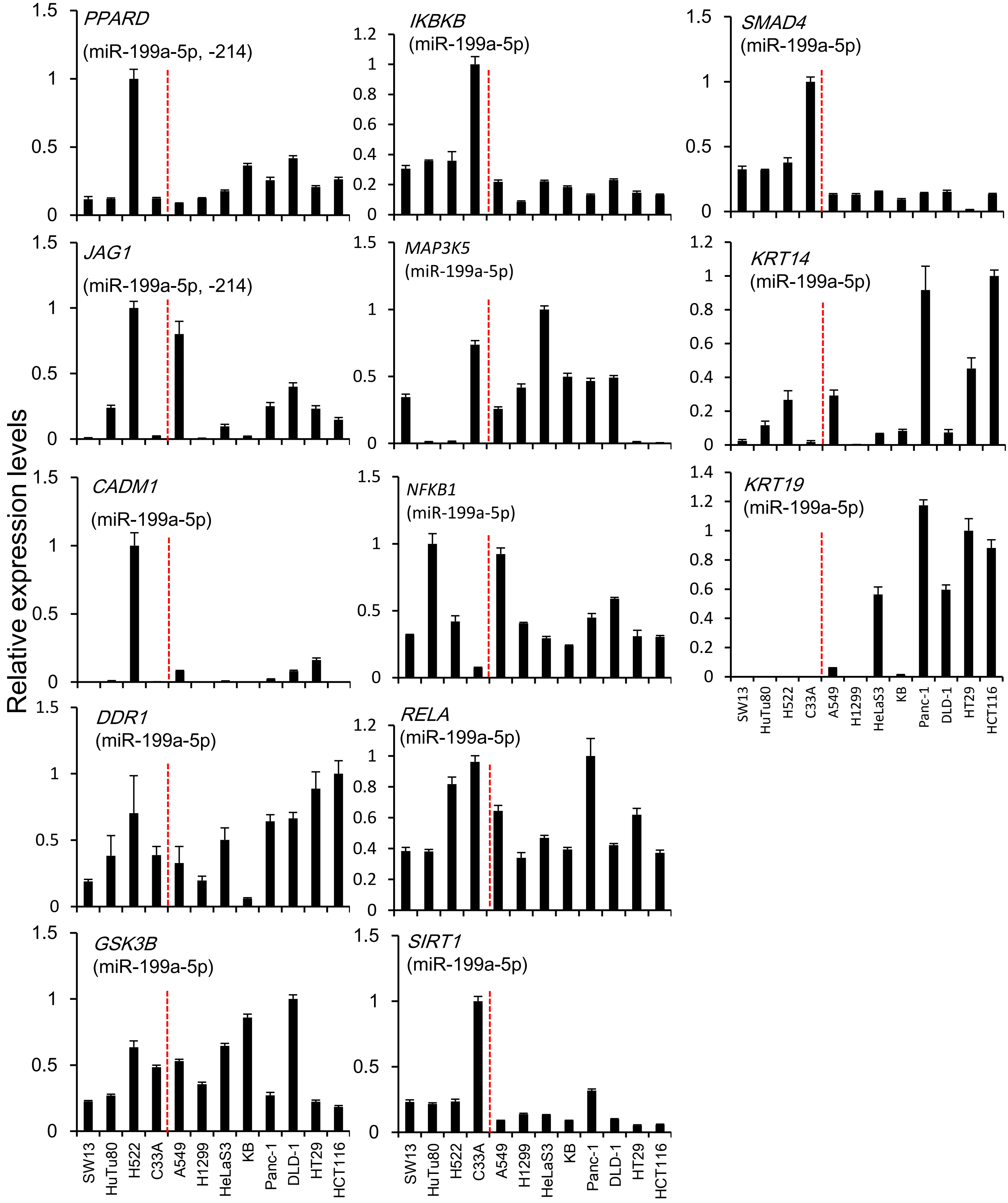

Supplementary Figure 2. mRNA expression profiles of each miR-199a-5p target gene in the epithelial tumor cell line panel, as determined by quantitative RT-PCR. The relative expression levels are shown by taking the highest levels as 1.0. Red break lines indicate the boundary between type 2 and type 1 cell lines.

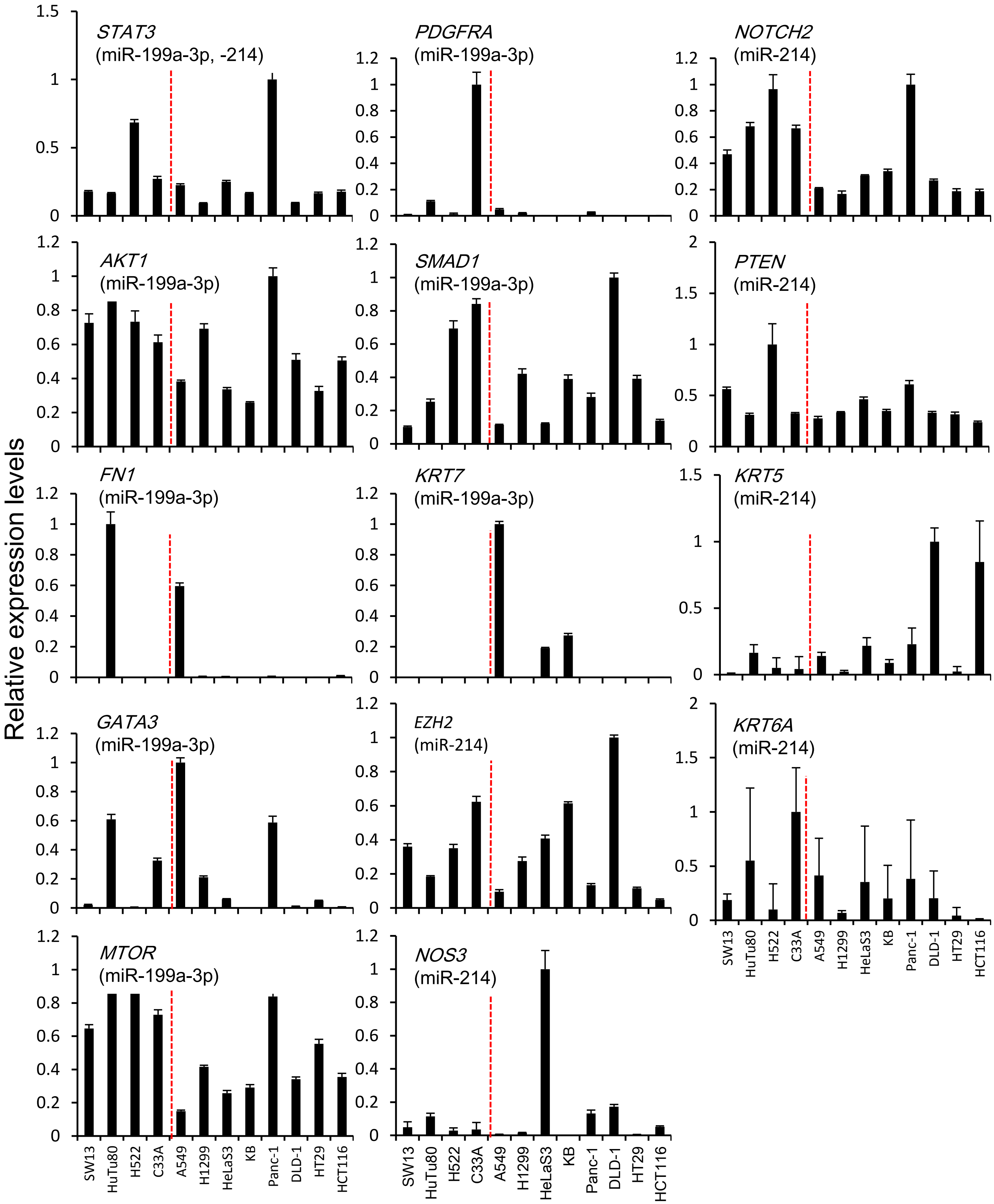

Supplementary Figure 3. mRNA expression profiles of each target gene for miR-199a-3p and miR-214 in the epithelial tumor cell line panel, as determined by quantitative RT-PCR. The relative expression levels are shown by taking the highest levels as1.0. Red break lines indicate the boundary between type 2 and type 1 cell lines.

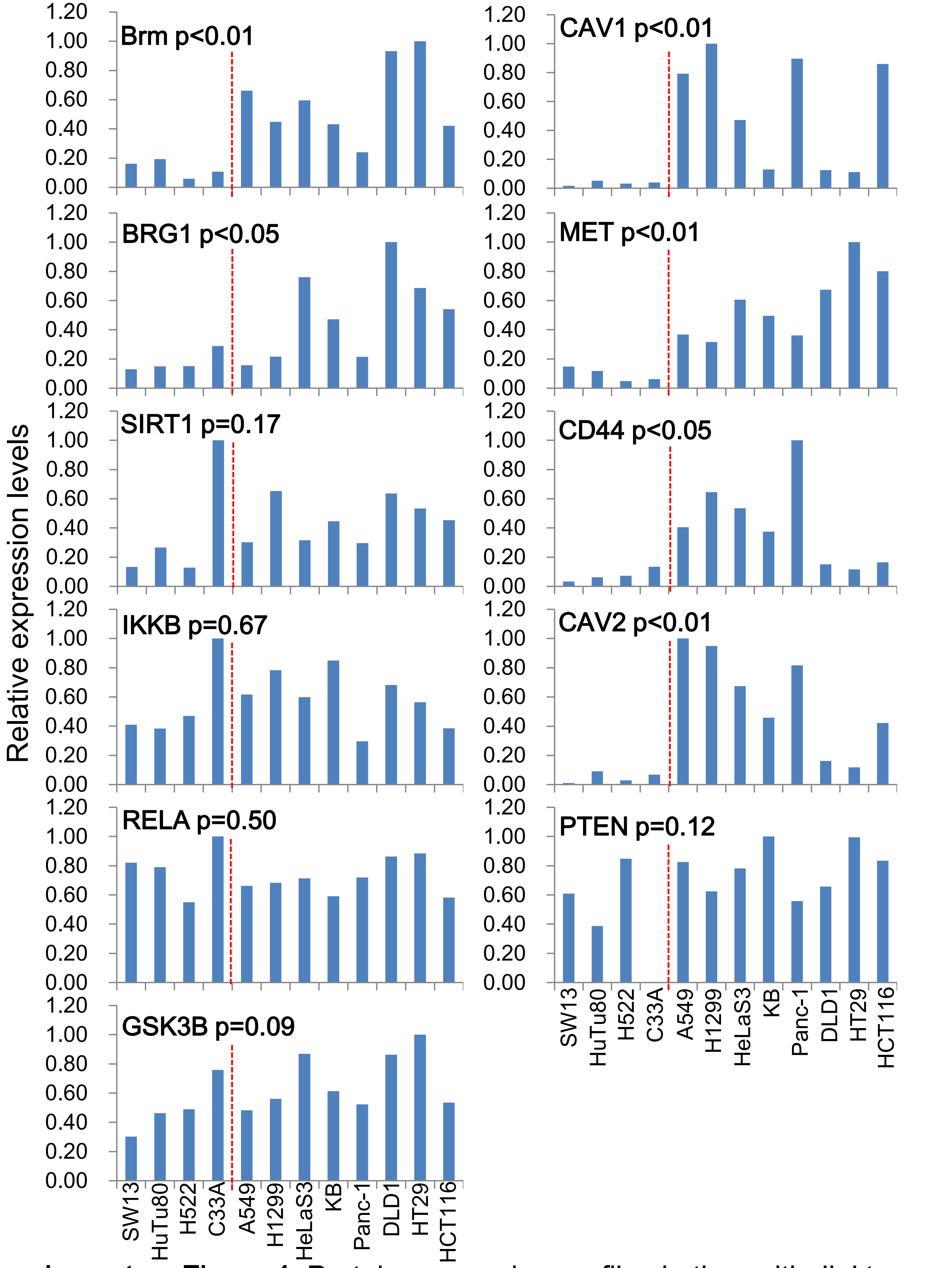

Supplementary Figure 4. Protein expression profiles in the epithelial tumor cell line panel. Western blots shown in Fig. 2b were used for quantification after normalization by β--actin bands (internal control). The relative expression levels are shown by taking the highest levels as1.0. Red break lines indicate the boundary between type 2 and type 1 cell lines.

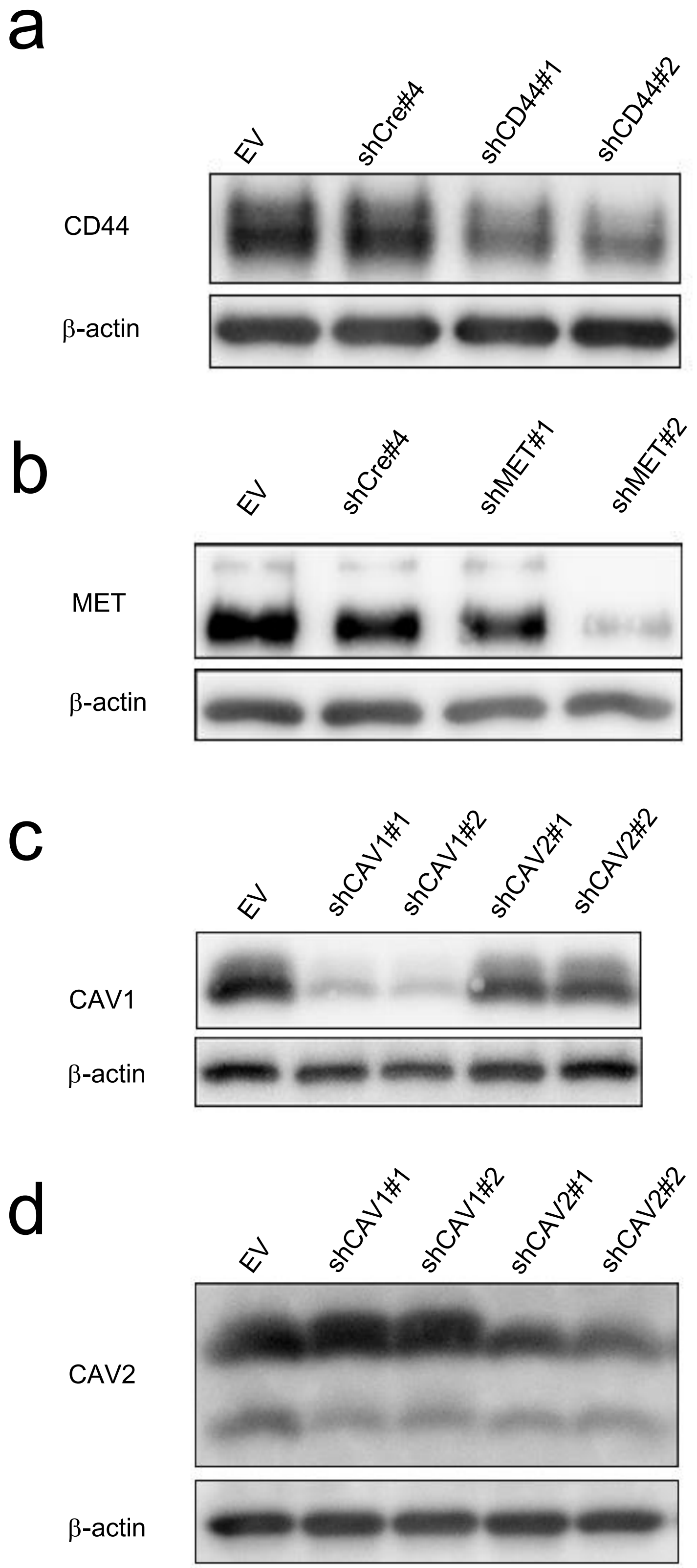

Supplementary Figure 5. Knockdown efficiency of shRNAs against CD44, MET, CAV1, and CAV2 were determined by western blot analysis of A549 cells. Cells were transduced with shRNA-expressing retroviral vectors against CD44 (a), MET (b), CAV1 (c) or CAV2(d), and Cre#4 (negative control), and total proteins were prepared and analyzed by western blotting using the corresponding antibody. EV=transduced with an empty vector (pSSSP).

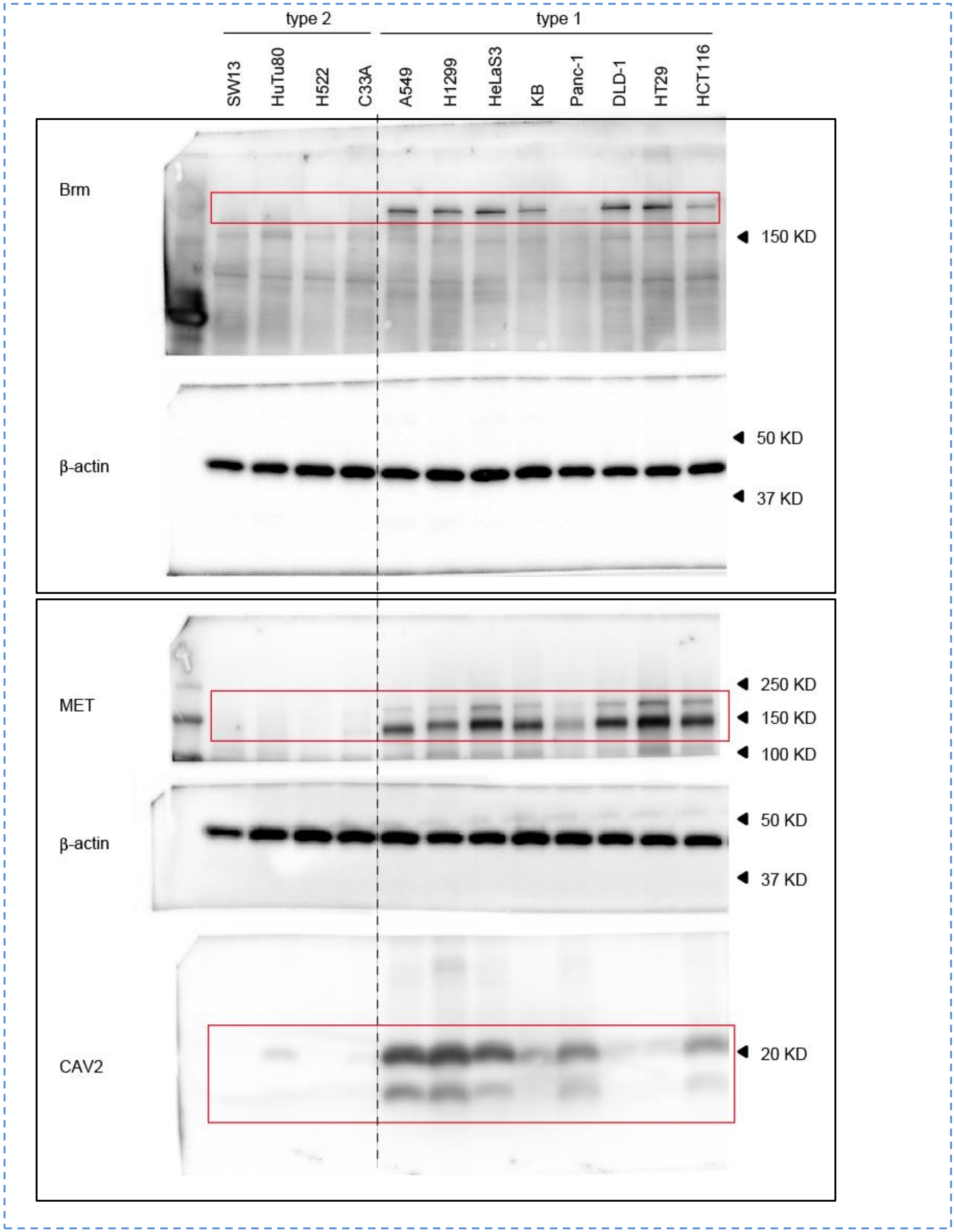

Supplementary Figure 6. Full-length images of the immunoblots. Red line boxes indicate the cropped images used in Figure 2b. β-actin was used as an internal control. Blots in a black line box are originated from the same gel. In gels in a blue broken line box, the same set of protein samples was charged. Arrowheads indicate the position of protein markers (#161-0374; BIO-RAD).

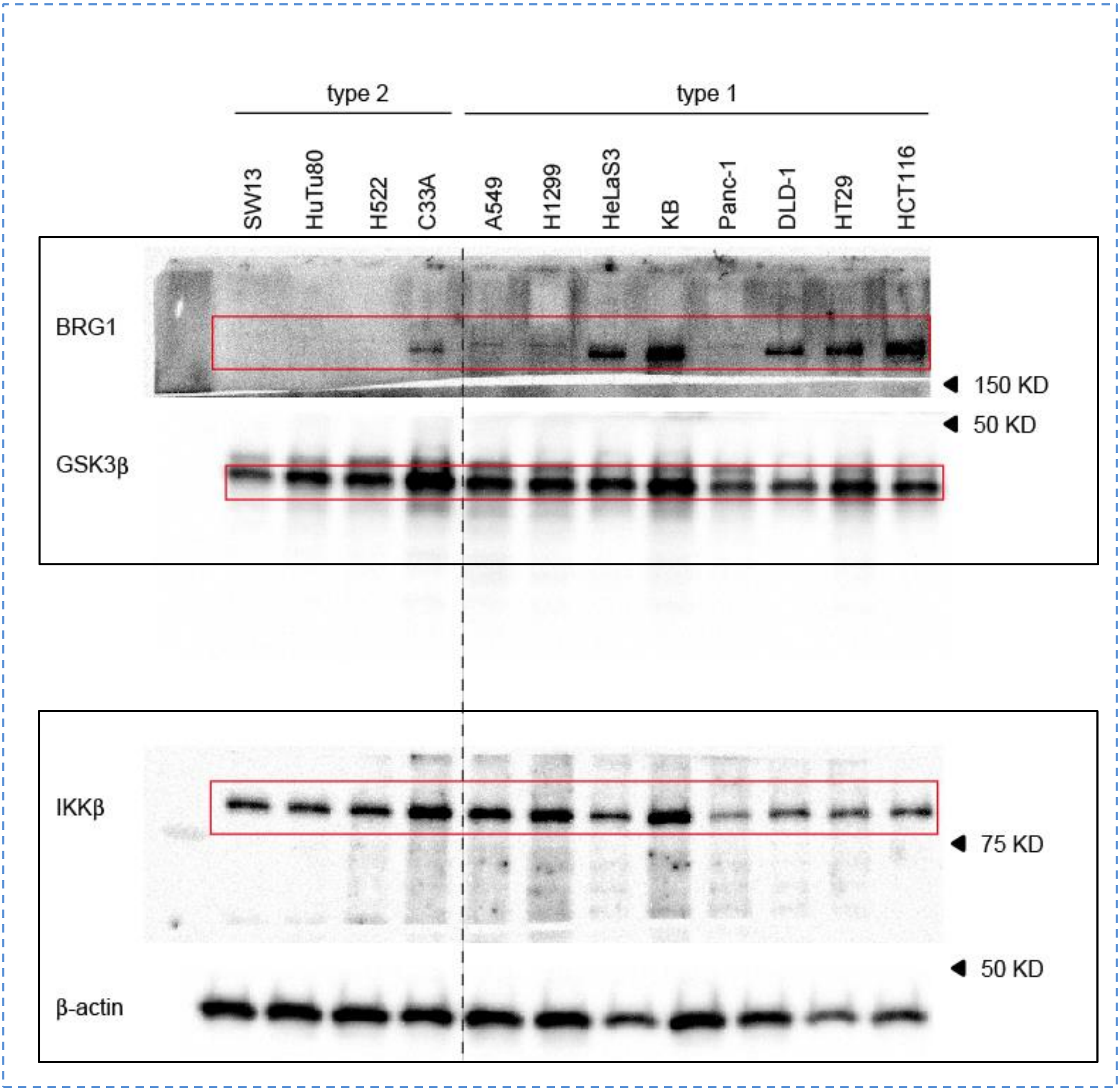

Supplementary Figure 6. Continued

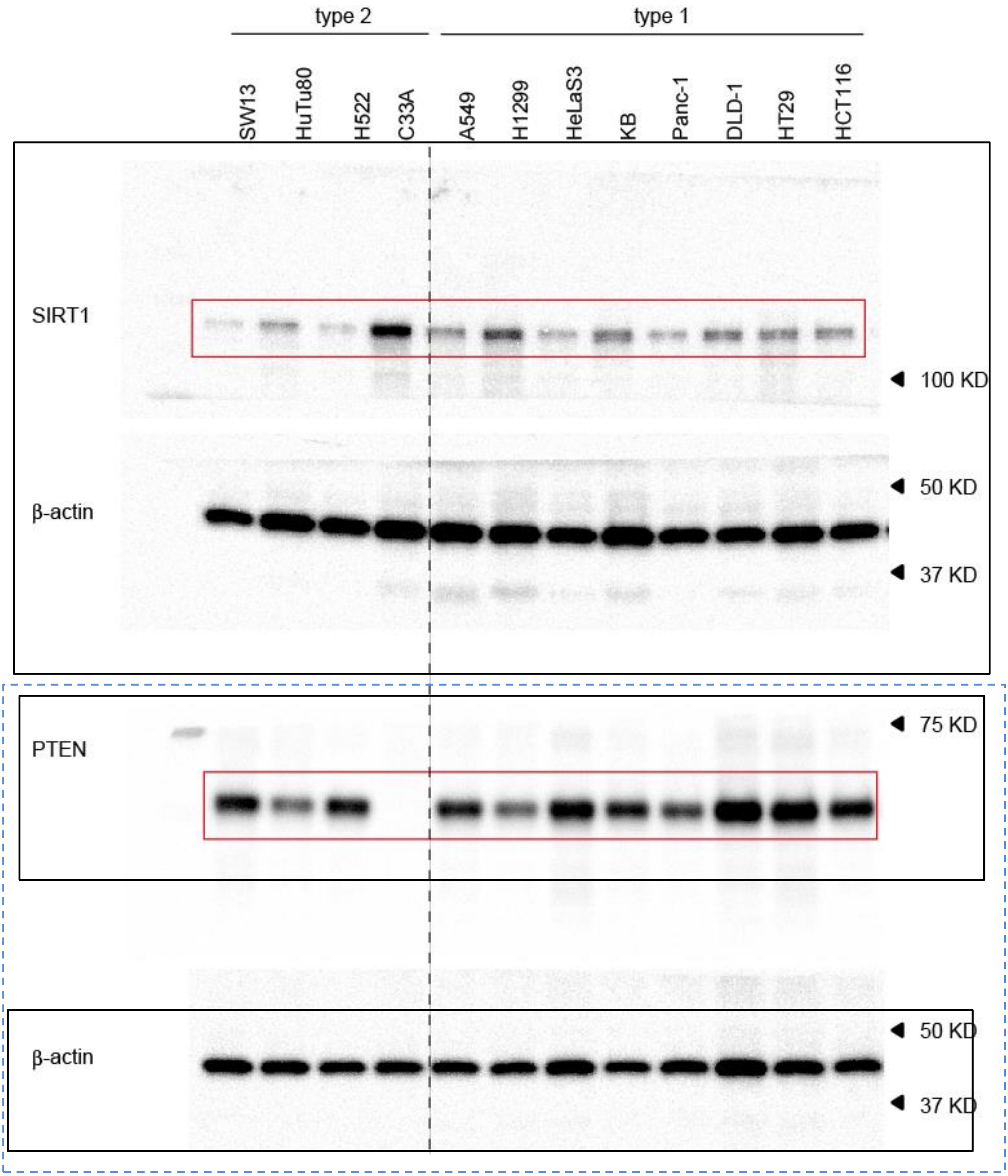

Supplementary Figure 6. Continued

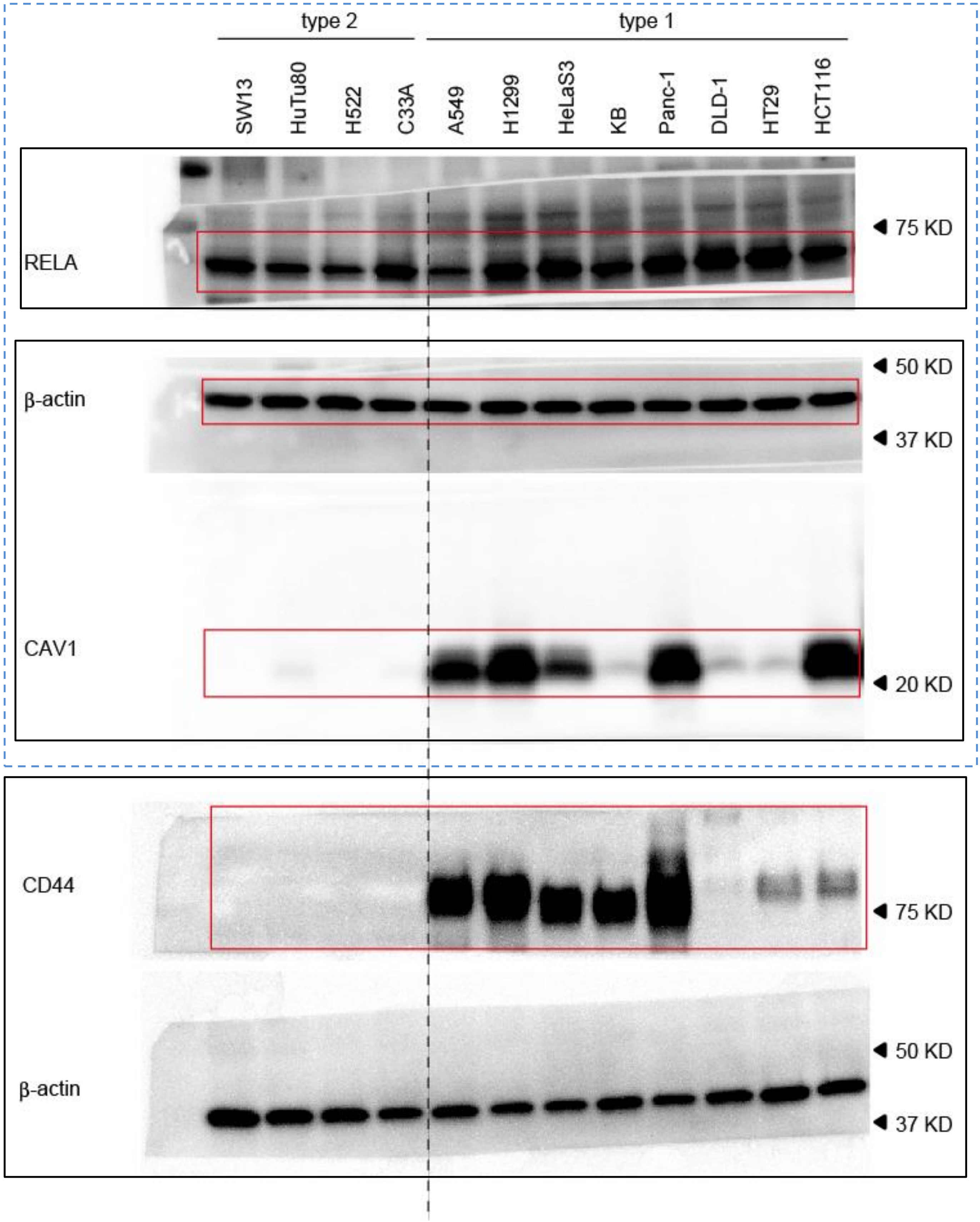

Supplementary Figure 6. Continued

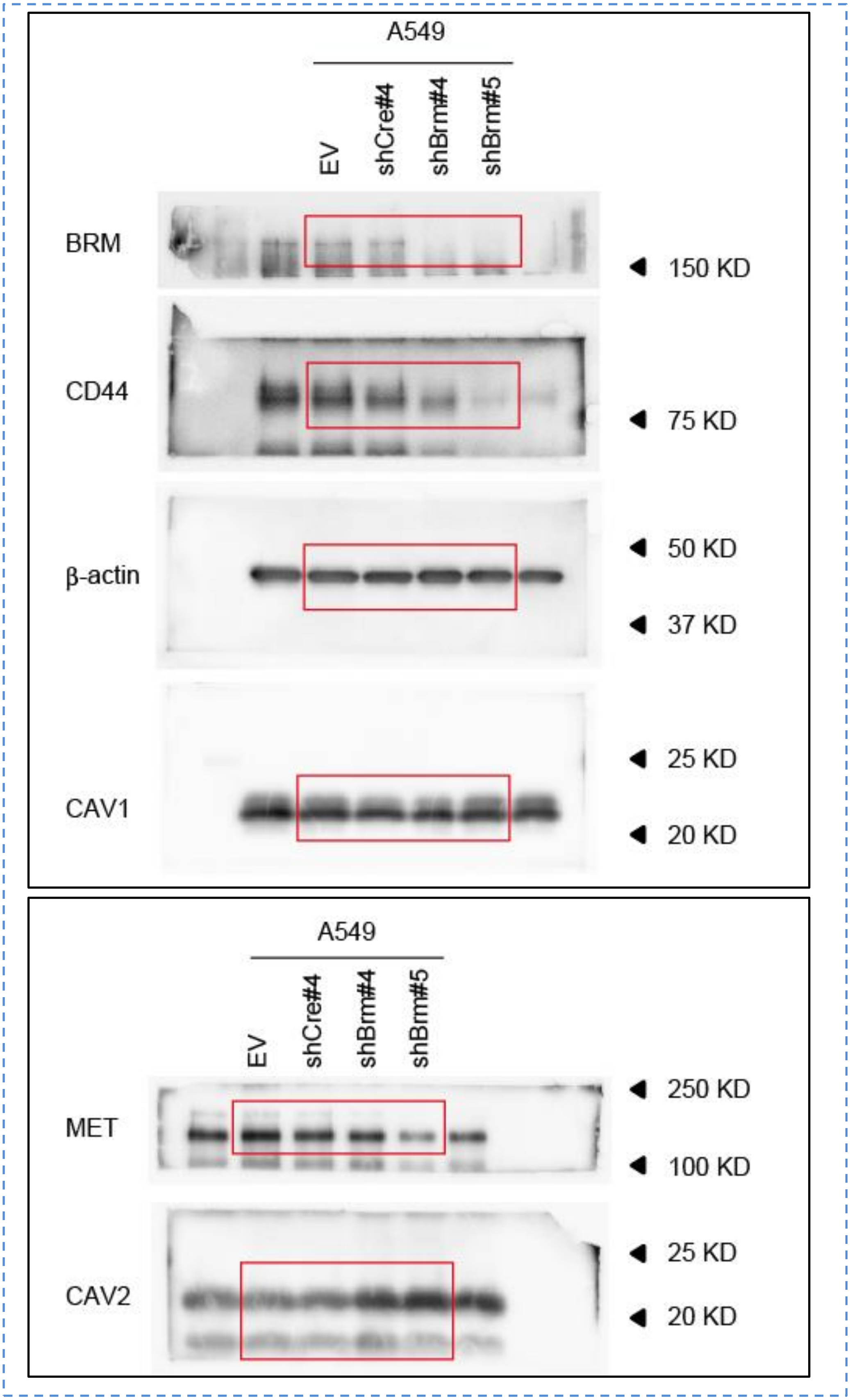

Supplementary Figure 7. Full-length images of the immunoblots. Red line boxes indicate the cropped images used in Figure 3c. β-actin was used as an internal control. Blots in a black line box are originated from the same gel. In gels in a blue broken line box, the same set of protein samples was charged. Arrowheads indicate the position of protein markers (#161-0374; BIO-RAD).

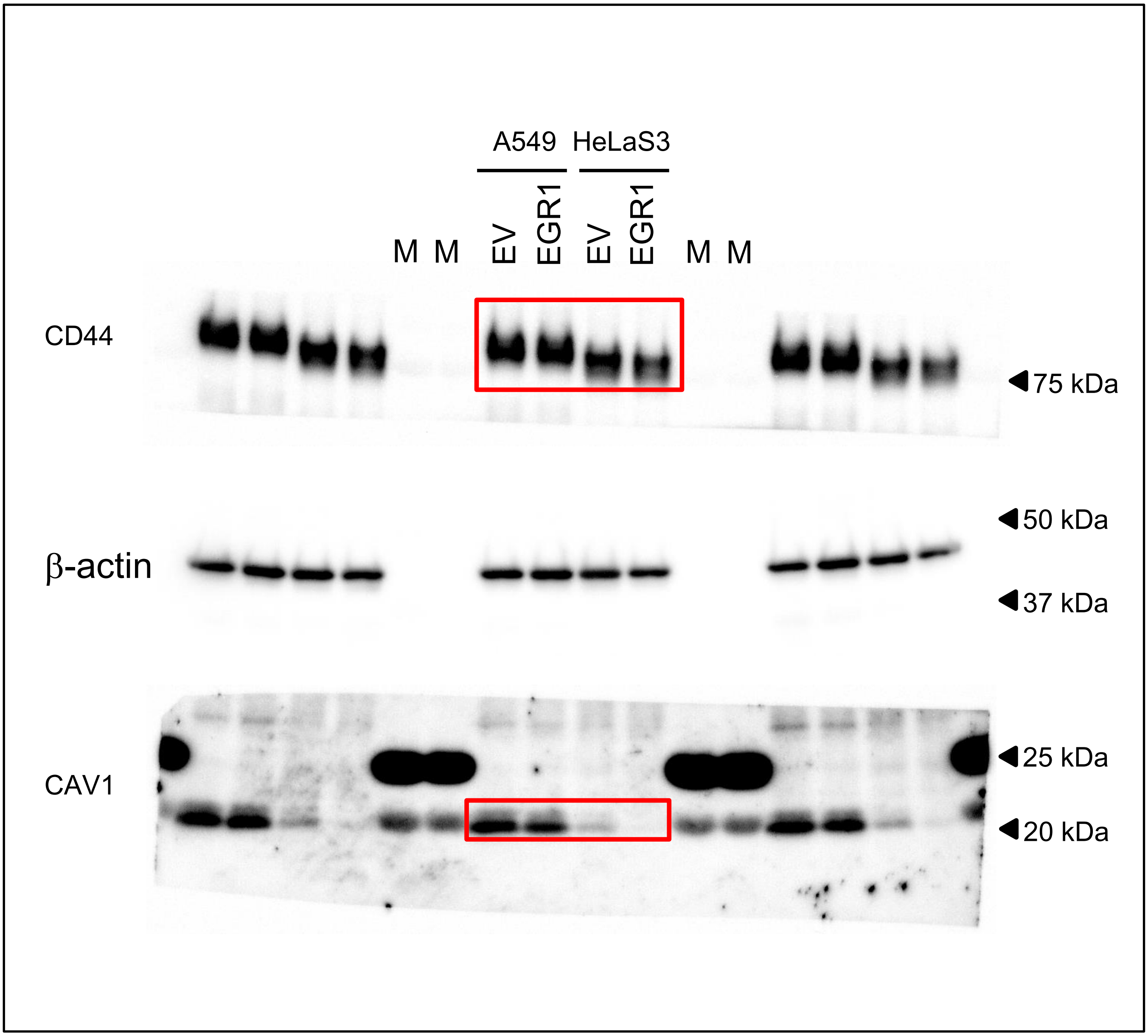

Supplementary Figure 8. Full-length images of the immunoblots. Red line boxes indicate the cropped images used in Figure 5b. β-actin was used as an internal control. Blots in a black line box are originated from the same gel. M means Protein size marker (#161-0374; BIO-RAD). Arrowheads indicate the position of marker proteins.

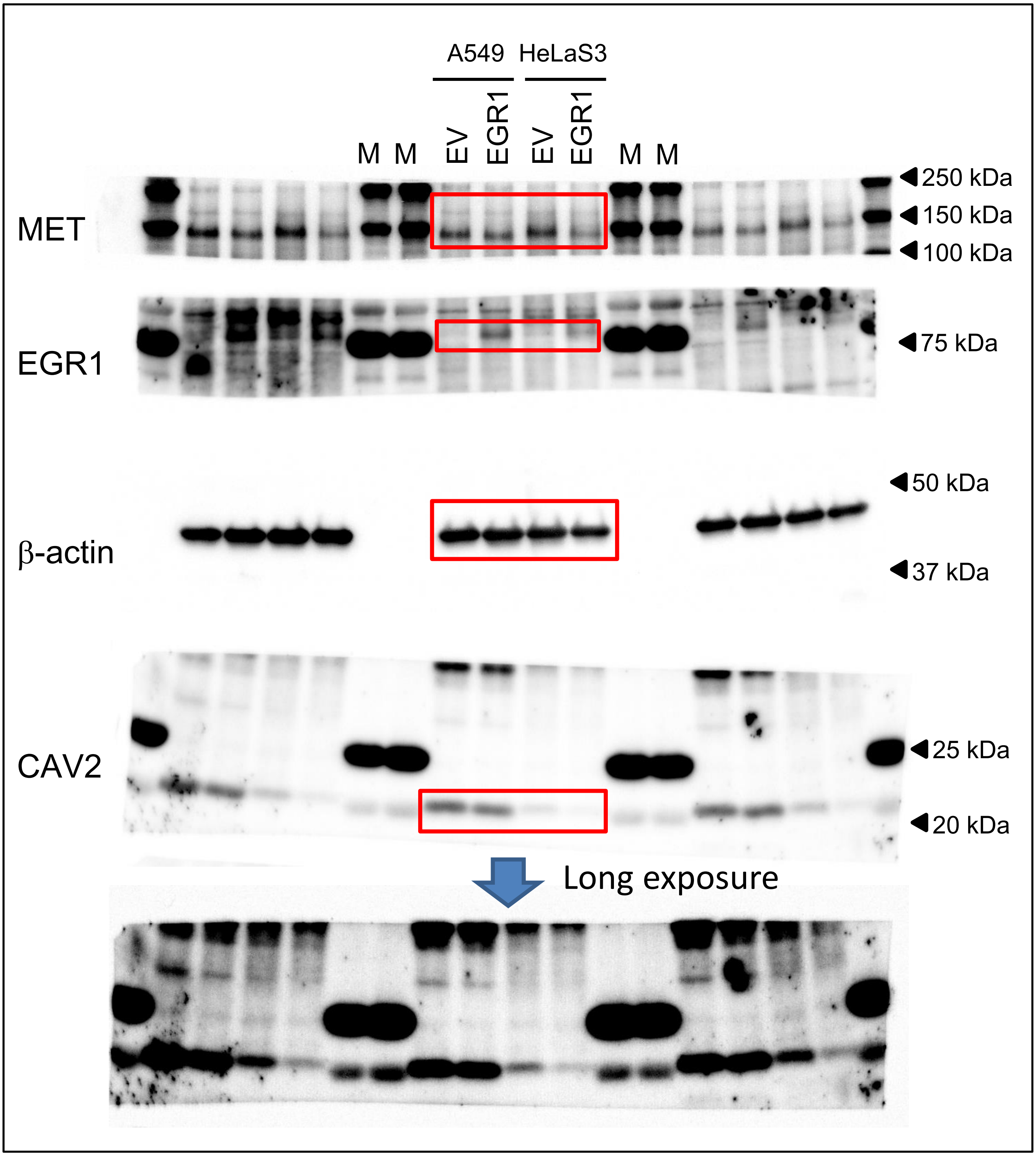

Supplementary Figure 8. Continued

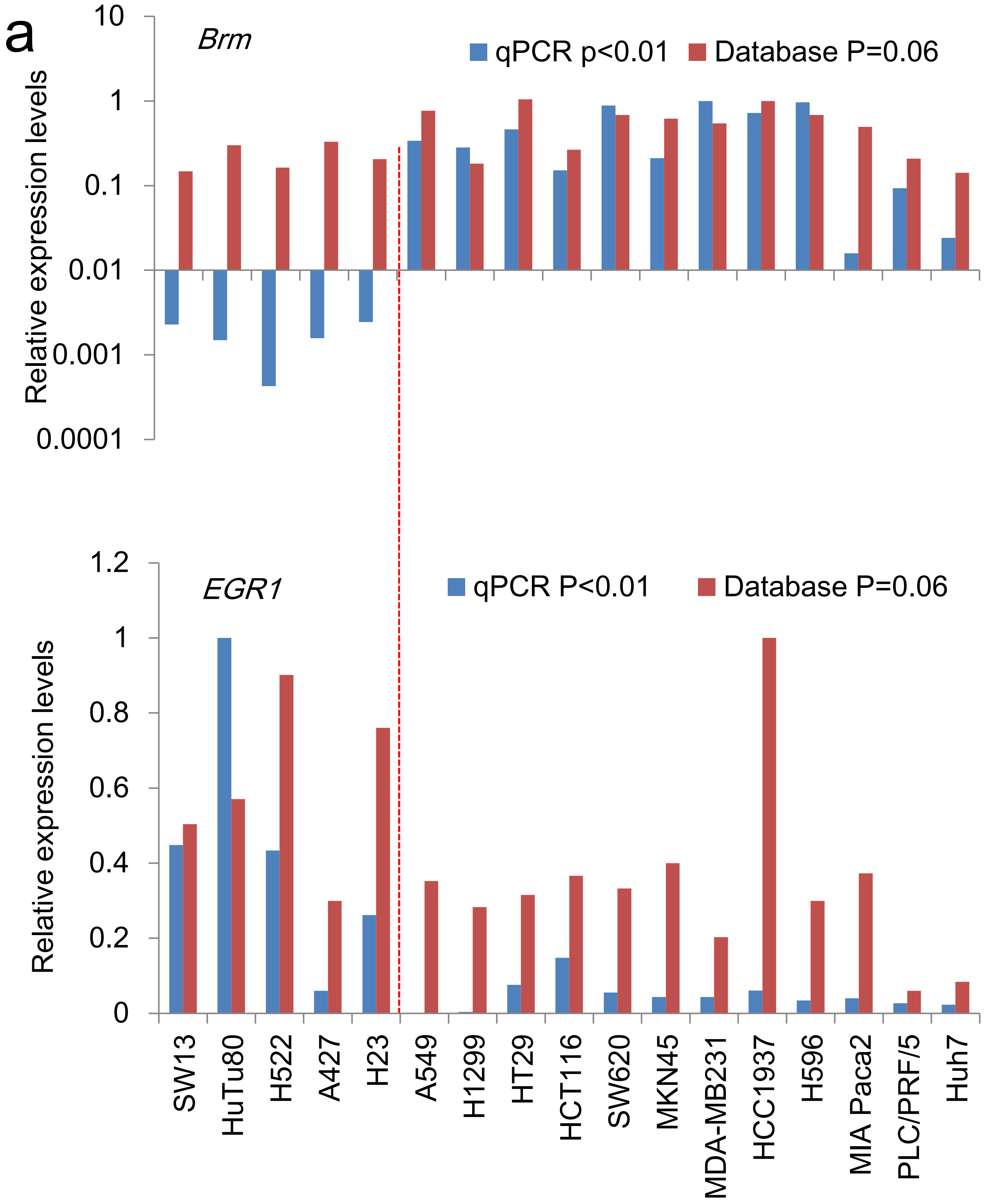

Supplementary Figure 9. Expression profiles of *Brm* and *EGR1* mRNA in 17 epithelial tumor cell line panel obtained from our quantitative PCR data using Brm-1 and EGR1-1 primer pairs (blue bars) and Sanger Database (red bars) . The each relative expression levels are shown by taking the highest levels as 1.0. Red break lines indicate the boundary between type 1 and type 2 cell lines.

Supplementary Table 1. List of the examined target genes of miR-199a-5p, -3p and miR-214

Gene	Accession Number	Reported miRNA to target Genes	a	b	c	d	Reference
PPARD	NM_006238	199a-5p, 214	×	×	×	×	Cell Metab 18, 341-54 (2013)
JAG1	NM_000214	199a-5p, 214	○	○	5p:○, 214:x	×	RNA Biol 9, 351-60 (2012)
KRT80	NM_182507	199a-5p, 214	×	×	○	×	-
CADM1	NM_014333	199a-5p	○	×	○	×	J Biol Chem 288, 11845-53 (2013)
DDR1	NM_001954	199a-5p	○	○	○	○	Mol Cancer 9, 227 (2010).
GSK3B	NM_002093	199a-5p	○	○	○	×	Cancer Lett 315, 189-97 (2012)
IKBKB	NM_001556	199a-5p	○	○	○	×	Oncogene 27, 4712-23 (2008)
MAP3K5	NM_005923	199a-5p	○	×	○	×	-
NFKB1	NM_003998	199a-5p	○	×	○	×	Am J Respir Crit Care Med 189, 263-73 (2014)
RELA	NM_021975	199a-5p	×	×	×	×	Am J Respir Crit Care Med 189, 263-73 (2014)
SIRT1	NM_012238	199a-5p	○	○	○	○	Circ Res 104, 879-86 (2009)
SMAD4	NM_005359	199a-5p	×	×	×	○	Nucleic Acids Res 40, 9286-97 (2012)
CAV1	NM_001753	199a-5p	○	○	○	○	PLoS Genet 9, e1003291 (2013)
KRT14	NM_000526	199a-5p	×	×	○	×	-
KRT19	NM_002276	199a-5p	×	×	×	×	Confirmed by our group
STAT3	NM_003150	199a-3p, 214	×	×	3p:×, 214:○	×	Mol Cancer Ther 10, 1337-45 (2011)
AKT1	NM_005163	199a-3p	×	×	×	○	-
FN1	NM_002026	199a-3p	×	○	○	×	-
GATA3	NM_002051	199a-3p	×	○	×	×	-
MTOR	NM_004958	199a-3p	×	×	○	○	Mol Cancer Ther 10, 1337-45 (2011)<br>Cancer Res 70, 5184-93 (2010)
PDGFRA	NM_006206	199a-3p	×	○	○	×	-
SMAD1	NM_005900	199a-3p	×	×	×	×	J Biol Chem 284, 11326-35 (2009)
CAV2	NM_001233	199a-3p	×	○	○	○	J Cell Sci 124, 2826-36 (2011)
CD44	NM_000610	199a-3p	×	×	○	○	Biochem Biophys Res Commun 403, 120-5 (2010)<br>FEBS J 279, 2047-59 (2012)
KRT7	NM_005556	199a-3p	×	×	×	○	Int J Cancer 125, 345-52 (2009)
MET	NM_000245	199a-3p	×	○	×	○	Mol Cancer Ther 10, 1337-45 (2011)<br>Cancer Res 70, 5184-93 (2010)
EZH2	NM_004456	214	×	×	×	○	Mol Cell 36, 61-74 (2009)
NOS3	NM_000603	214	×	×	○	×	Eur J Pharm Sci 38, 370-7 (2009)
NOTCH2	NM_024408	214	×	×	○	×	-
PTEN	NM_000314	214	○	×	○	○	Cancer Res 68, 425-33 (2008)
KRT5	NM_000424	214	○	×	○	×	-
KRT6A	NM_005554	214	×	×	○	×	-

a. mirna.org : (http://www.microrna.org/microrna/home.do), b. PicTar : (http://pictar.mdc-berlin.de/) c. TargetScan : (http://www.targetscan.org/), d. miRTarBase : (http://mirtarbase.mbc.nctu.edu.tw/)

Supplementary Table 2. Relative protein expression levels of EGR1, CD44, MET, CAV1 and CAV2 after EGR1 introduction into A-549 or HeLaS3. Three western blots (one of them was shown in Fig5b) were used for quantification after normalization by β-actin (internal control). The protein bands of cells transduced with the empty vector (EV) were taken as 1.0 and P-values were calculated by the student's t-test (n=3).

	A549		HeLaS3	
Protein	Relative expression levels	P value	Relative expression levels	P value
EGR1	2.16±0.62	p<0.001	1.91±0.25	p<0.001
CD44	0.96±0.11	p=0.42	1.06±0.07	p=0.07
MET	075±0.03	p<0.01	0.80±0.11	p<0.05
CAV1	0.77±0.06	p<0.001	0.61±0.12	p<0.001
CAV2	0.76±0.12	p<0.01	0.62±0.13	p<0.001

Supplementary Table3. Epithelial tumor cell lines used in this study and their classification

Cell lines used in Fig.1a as the original panel are shown in blue. Cell lines that are newly added in Fig.7 are shown in white. For *Brm* and *EGR1* mRNA, qPCR data using primer pairs Brm-1 and EGR1-1 were used (Fig. 7). The relative expression levels are shown by taking the highest levels as 1.0. Criteria for classification of three expression levels (+,±, -) are summarized in the upper part.

	Brm	EGR1	miR-199a-3p
+	0.10<	0.15<	0.10≦
±	0.02-0.10	0.06-0.15	-
-	<0.02	<0.06	<0.10

Type	Cell line	Origin	Brm	EGR1	199a-3p
2	SW13	Adrenal carcinoma	0.00	0.45	0.17
2	HuTu80 (AZ521)	Duodenum adenocarcinoma	0.00	0.99	0.32
2	NCI-H522	NSCLC	0.00	0.43	1.00
2	C33A	Cervical carcinoma	0.00	1.00	0.08
2	A427	Lung carcinoma	0.00	0.06	0.17
2	H23	NSCLC	0.00	0.26	0.11
1	A549	Lung carcinoma	0.34	0.00	0.00
1	H1299	NSCLC	0.28	0.00	0.00
1	HT29	Colorectal adenocarcinoma	0.46	0.08	0.00
1	HCT116	Colorectal adenocarcinoma	0.15	0.15	0.05
1	HeLaS3	Cervical carcinoma	0.44	0.00	0.00
1	KB	Cervical carcinoma	0.40	0.00	0.00
1	Panc-1	Pancreatic carcinoma	0.15	0.00	0.01
1	DLD-1	Colorectal adenocarcinoma	0.58	0.13	0.00
1	SW620	Colorectal adenocarcinoma	0.89	0.05	0.04
1	MKN45	Gastric adenocarcinoma	0.21	0.04	0.01
1	MDA-MB231	Mammary adenocarcinoma	1.00	0.04	0.00
1	SUM149PT	Ductal carcinoma	0.37	0.02	0.01
1	HCC1937	Ductal carcinoma	0.72	0.06	0.03
1	H596	Lung adenosquamous carcinoma	0.96	0.03	0.01
1	MIA Paca2	Pancreatic carcinoma	0.02	0.04	0.01
1	PLC/PRF/5	Hepatoma	0.09	0.03	0.01
1	Huh7	Hepatoma	0.02	0.02	0.01
3	MCF7	Mammary adenocarcinoma	0.78	0.19	0.04
3	GCIY	Stomach carcinoma	0.01	0.04	0.04
3	MKN1	Stomach adenosquamous carcinoma	0.31	0.79	0.03

Supplementary Table 4. List of primer pairs used for qPCR and oligonucleotides used for shRNA expression vector construction.

Gene	F	R	*Gene*	F	R
KRT80	tcagctgaagaaggacctgg	caactccacgaagctctcca	*CD44v*	tctttcaatgacaacgcagca	ttgggtctcttcttccacctg
CADM1	cgtgacagtgatcgagggag	tttcctgtgggggatcggta	*KRT7*	aatgagtttgtggtgctgaagaag	gtcaactccgtctcattgaggg
DDR1	gctggaaggaccgctgg	agtcgggcaaccatgggg	*MET*	atgtgagatgtctccagcatttt	gcaaagctgtggtaaactctgtt
GSK3B	tagtcgagccaaacagacgc	tccaacaagaggttctgcgg	*EZH2*	ctgcttcctacatcgtaagtgc	tgagagcagcagcaaactcc
IKBKB	agactcagatctccccacgg	ctgctgagacatggaagcca	*NOS3*	gagtatgacgtggtgtccctc	tccatcagggcagctgcaaa
MAP3K5	accgggacataaagggtgaca	tatgccagcaagcctctttga	*NOTCH2*	agcactcaggtgtctgcatc	ttctggcagggctgattctg
NFKB1	ttctggaccgcttgggtaac	aatggcattcagaccgtccc	*PTEN*	agtggcggaacttgcaatcctca	tcccgtcgtgtgggtcctgaa
RELA	cctcctgttgtctcgaaccc	tgccttttgcttcccaatcg	*KRT5*	tggagggcgaggaatgcag	catatccagaggaaacactgcttg
SIRT1	tcagtgtcatggttcctttgc	gttcatcagctgggcaccta	*KRT6A*	ttggtaaagcccagcccttcc	agcaggactaggaatcaggctc
SMAD4	ctggcctgttcacaatgagc	tgtgcaaccttgctctctca	*Brm-1*	acaaagggaaaggcaagaaaag	gtcccacttccttctgactgtt
CAV1	cgcgaccctaaacacctcaa	gccgtcaaaactgtgtgtcc	*Brm-2*	taagagtgcccggcagaaaa	gagcttaattttcaccttgactga
KRT14	aggagatcgccacctaccg	cacatctctggatgactgcga	*Brm-3*	cagggcgacagctcagtgaa	ggctccggtacttatgattacga
KRT19	gcgactacagccactactacac	aatcctggagttctcaatggtg	*Brm-4*	tgaccaaatgggcctcaaaga	aaccaggcacaatcaaaccg
STAT3	cccttggattgagagtcaaga	aagcggctatactgctggtc	*EGR4*	tcttcaacctcatgtcgggc	gatccggggagtaaaggtcc
AKT1	ggcaaggtgatcctggtgaa	ggtcgtgggtctggaaagag	*BRG1*	gagtgacgatgacagtgaggag	atgccatctcagctctggac
FN1	agtggaagtgtgagaggcac	tgaggctgcggttggtaaac	*EGR1-1*	agcagcagcagcaccttc	tctcgttgttcagagagatgtca
GATA3	ctcttcgctacccaggtgac	acgactctgcaattctgcga	*EGR1-2*	ccttcaaccctcaggcggac	agcggccagtataggtgatg
MTOR	caggctggctcttgctcata	ggtcacctgagggtgaactg	*EGR1-3*	ctacgagcacctgaccgc	agtggtttggctggggtaac
PDGFRA	gtctggagcgtttgggaaggt	gatctggccgtgggttttagc	*EGR1-4*	gggacatgctcacctctagc	tctggagaaccgaagctcag
SMAD1	cacccgtttcctcactctcc	aaccgcctgaacatctcctc	*EGR2*	ctttgaccagatgaacggagt	gtctggtttctaggtgcagagac
CAV2	ctcgcatctcaagctgggct	tgaaggcagaaccattaggca	*EGR3*	agagaaatgcctcggatgcc	agatcaaactgccctgaggc
CD44	tggcgcagatcgatttgaata	ccgtccgagagatgctgtag	*EGR4*	tcttcaacctcatgtcgggc	gatccggggagtaaaggtcc

shRNA	sense	antisense
Brm#5	tttgaagaaatgtggataaagatcgcttcctgtcacgatctttatccacatttcttcttttttg	aattcaaaaaagaagaaatgtggataaagatcgtgacaggaagcgatctttatccacatttctt
CD44#1	tttgaggaaacatttgacttatctgcttcctgtcacagataagtcaaatgtttcctcttttttg	aattcaaaaaagaggaaacatttgacttatctgtgacaggaagcagataagtcaaatgtttcct
CD44#2	tttgcccattgttcattcttgtgcgcttcctgtcacgcacaagaatgaacaatgggcttttttg	aattcaaaaaagcccattgttcattcttgtgcgtgacaggaagcgcacaagaatgaacaatggg
MET#1	tttgccactcatttagaattctaggcttcctgtcacctagaattctaaatgagtggcttttttg	aattcaaaaaagccactcatttagaattctaggtgacaggaagcctagaattctaaatgagtgg
MET#2	tttgtaatttgttgataaatatttgcttcctgtcacaaatatttatcaacaaattacttttttg	aattcaaaaaagtaatttgttgataaatatttgtgacaggaagcaaatatttatcaacaaatta
CAV1#1	tttggtaatttgagagaaatatgagcttcctgtcactcatatttctctcaaattaccttttttg	aattcaaaaaaggtaatttgagagaaatatgagtgacaggaagctcatatttctctcaaattac
CAV1#2	tttggaataagttcaaattcttctgcttcctgtcacagaagaatttgaacttattccttttttg	aattcaaaaaaggaataagttcaaattcttctgtgacaggaagcagaagaatttgaacttattc
CAV2#1	tttgctttagtacaatagtatacagcttcctgtcactgtatactattgtactaaagcttttttg	aattcaaaaaagctttagtacaatagtatacagtgacaggaagctgtatactattgtactaaag
CAV2#2	tttgctaataagtgacaaataagagcttcctgtcactcttatttgtcacttattagcttttttg	aattcaaaaaagctaataagtgacaaataagagtgacaggaagctcttatttgtcacttattag
